# Supplementary material for: Small molecules targeting the disordered transactivation domain of the androgen receptor induce the formation of collapsed helical states
Source: Nat Commun. 2022 Oct 27;13:6390. doi: 10.1038/s41467-022-34077-z (PMC9613762; doi:10.1038/s41467-022-34077-z)
Supplement: Supplementary file 1 — Supplementary Information [file 41467_2022_34077_MOESM1_ESM.pdf]

Supplementary Information

Small Molecules Targeting the Disordered Transactivation Domain of the Androgen Receptor Induce the Formation of Collapsed Helical States

Jiaqi Zhu[1], Xavier Salvatella[2,3] and Paul Robustelli[1†]

[1]Dartmouth College, Department of Chemistry, Hanover, NH, 03755

[2]Institute for Research in Biomedicine (IRB Barcelona), The Barcelona Institute of Science and Technology, Baldiri Reixac 10, 08028

[3]ICREA, Passeig Lluís Companys 23, 0810 Barcelona, Spain

† To whom correspondence should be addressed.

Paul Robustelli

E-mail: Paul.J.Robustelli@Dartmouth.edu

MD Simulation Convergence Analyses

Convergence of the Tau-5_{R2_R3} REST2 MD simulation was assessed by a comparison of the secondary structure profiles, free energy surfaces of Tau-5_{R2_R3} conformations as a function of the alpha helical order parameter Sα (see main text methods) and the radius of gyration (R_g) and the intramolecular contact probabilities for each solute temperature rung in the REST2 temperature ladder (Fig. S1-S3). The relatively smooth solute-temperature dependence of each of these properties suggests the simulations are reasonably well converged. The same analyses were also performed on this simulation using demultiplexed replicas, which follow each independent replica through temperature space to determine if any individual replicas became stuck in local minima as they diffuse through the temperature ladder (Fig. S4-S6). We also found the statistical fluctuations to be relatively well converged among demultiplexed replicas; with only 3 of replicas (replicas 6, 11 and 12) showing substantial deviations from the average contact probabilities and secondary structure profiles relative to the remaining 13 replicas.

The helical propensity, intramolecular contact probabilities, and free energy surfaces of each replica as a function of R_g and the α-helical order parameter Sα are shown for each temperature replica of a REST2 simulation of Tau-5_{R2_R3} in the presence of EPI-002 in Fig. S7-S9 and for each demultiplexed replica in Fig. S10-S12. We observed that the helical propensities decrease relatively smoothly and continuously across temperature replicas as the solute temperature increases, and the demultiplexed replicas have similar helical propensity profiles, with helical elements localized to the same region and differing only in magnitude between replicas. The helical propensity, contact probabilities, and free energy surfaces of each replica are shown for each temperature replica of a REST2 simulation of Tau-5_{R2_R3} in the presence of EPI-7170 in Fig. S13-S15 and for each demultiplexed replica in Fig. S16-S18. The smoothly varying α-helical and β-sheet propensities across temperature replicas and the similarity of helical propensities, intramolecular contact probabilities, and free energy surfaces as function of R_g and Sα of the demultiplexed replicas suggest that simulations of Tau-5_{R2_R3} + EPI-7170 are exceptionally well converged.

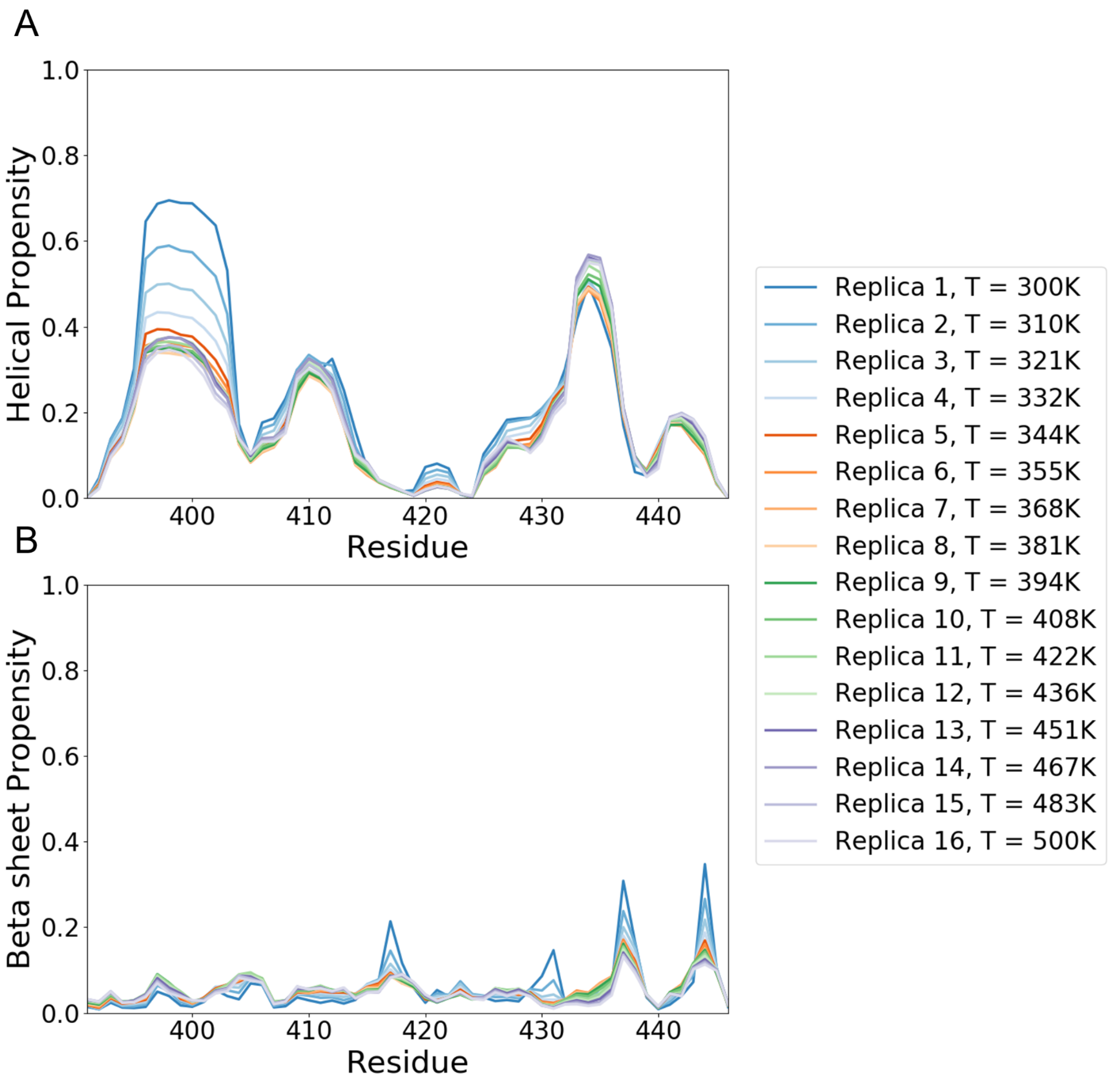

Supplementary Figure 1. **Secondary structure propensities observed in REST2 MD simulations of apo Tau-5$_{R2_R3}$.** Comparison of α-helical (A) and β-sheet (B) propensities observed in the 16 temperature rungs of an apo Tau-5$_{R2_R3}$ REST2 MD simulation. Secondary structure content is calculated by the DSSP algorithm. The solute temperature of each replica is shown in the figure legend.

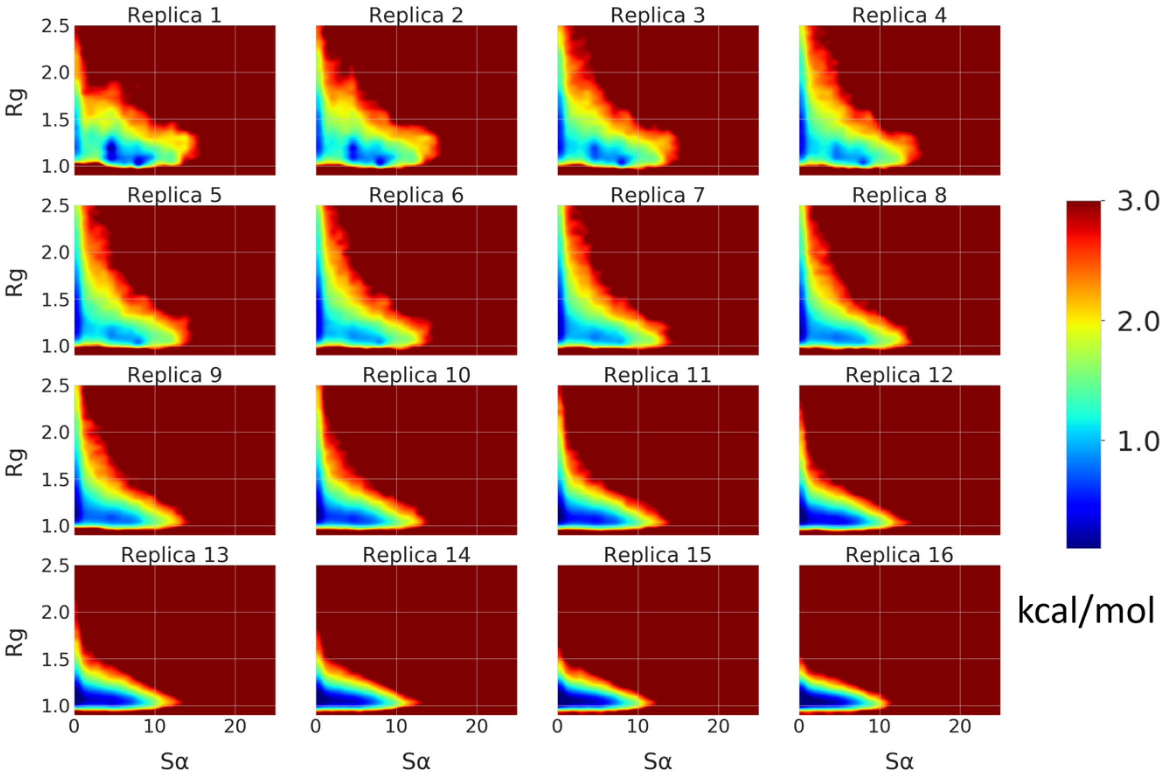

Supplementary Figure 2. Free energy surfaces from REST2 MD simulations of apo Tau-5$_{R2_R3}$. Comparison of free energy surfaces of the 16 solute temperature rungs of an apo Tau-5$_{R2_R3}$ REST2 MD simulation as a function of the α-helical order parameter Sα and radius of gyration (R_g). R_g is reported in nm.

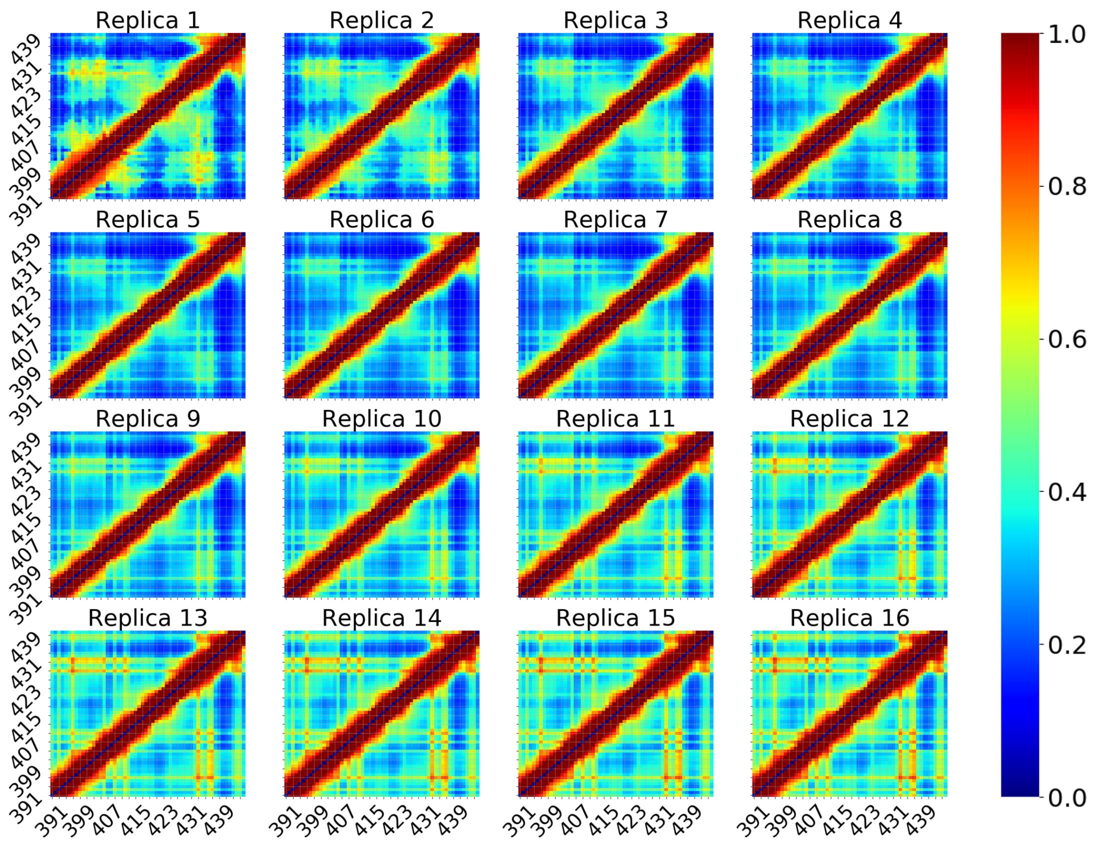

Supplementary Figure 3. Intramolecular contact maps from REST2 MD simulations of apo Tau-5$_{R2_R3}$. Comparison of the intramolecular contact probabilities observed in the 16 solute temperature rungs of an apo Tau-5$_{R2_R3}$ REST2 MD simulation. Contacts between two residues are defined using a distance cutoff of 12Å between Cα atoms.

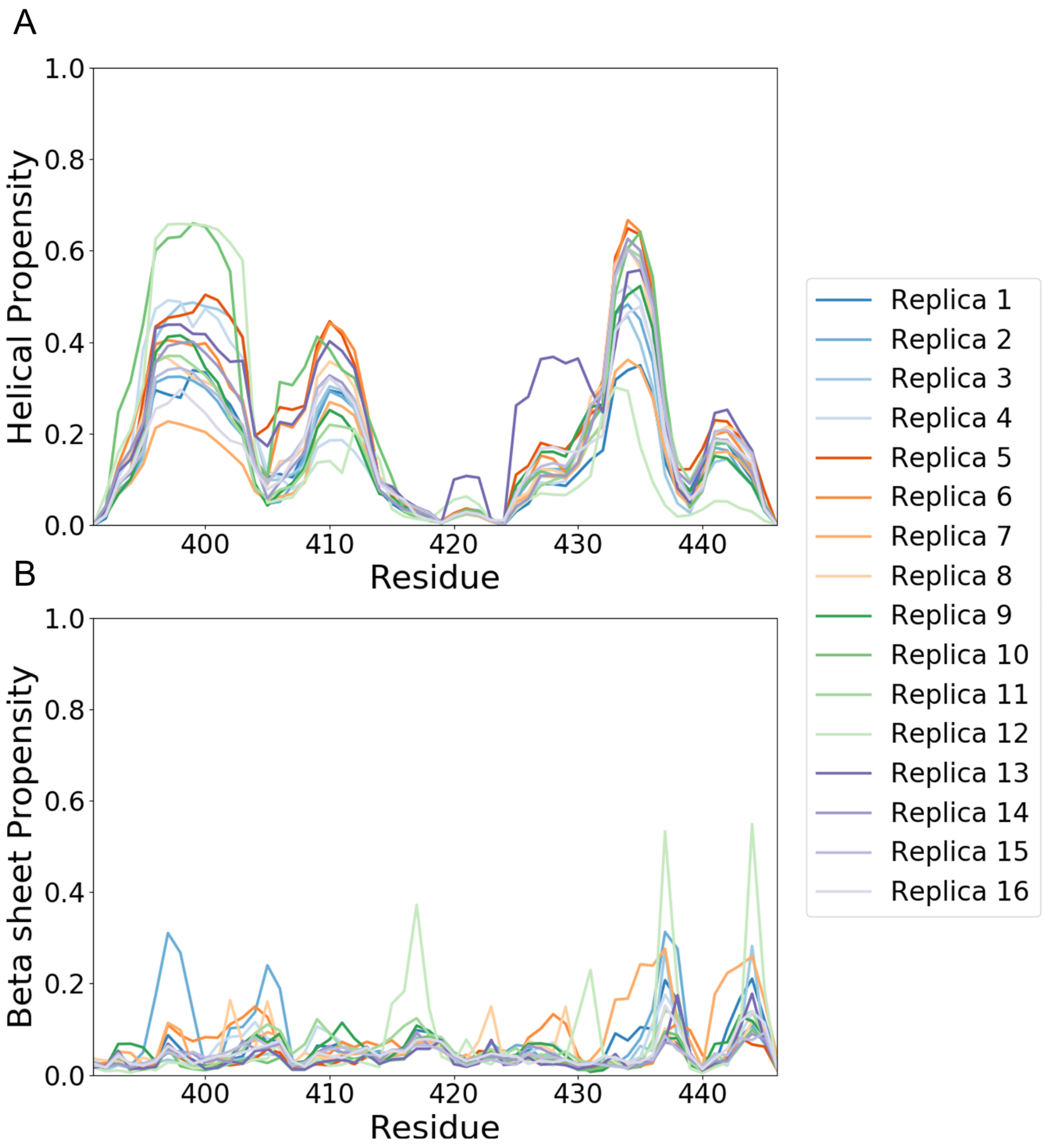

Supplementary Figure 4. Secondary structure propensities observed in demultiplexed replicas from REST2 MD simulations of apo Tau-5$_{R2_R3}$. Comparison of α-helical (A) and β-sheet (B) propensities observed in the 16 independent demultiplexed replicas of an apo Tau-5$_{R2_R3}$ REST2 MD simulation. Secondary structure content is calculated by the DSSP algorithm. Replicas 6, 11, and 12 show the largest deviations in secondary structure propensity from the average helical propensity across all demultiplexed replicas, but still contain helical propensities in the same regions, differing only in relative populations

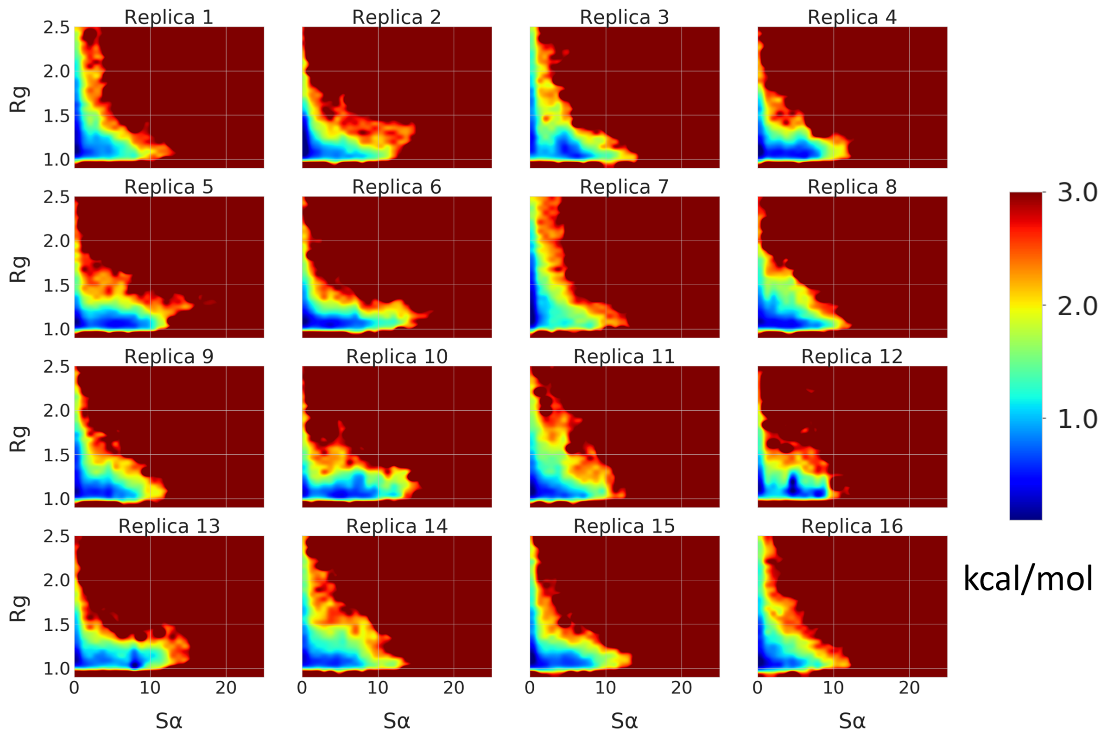

Supplementary Figure 5. Free energy surfaces of demultiplexed replicas from REST2 MD simulations of apo Tau-5$_{R2_R3}$. Comparison of free energy surfaces of Tau-5$_{R2_R3}$ conformations as a function of the α-helical order parameter Sα and radius of gyration (R_g) for the 16 demultiplexed replicas of an apo Tau-5$_{R2_R3}$ REST2 MD simulation. R_g is reported in nm. Replica 7 samples conformations with substantially smaller Sα values than the other demultiplexed replicas.

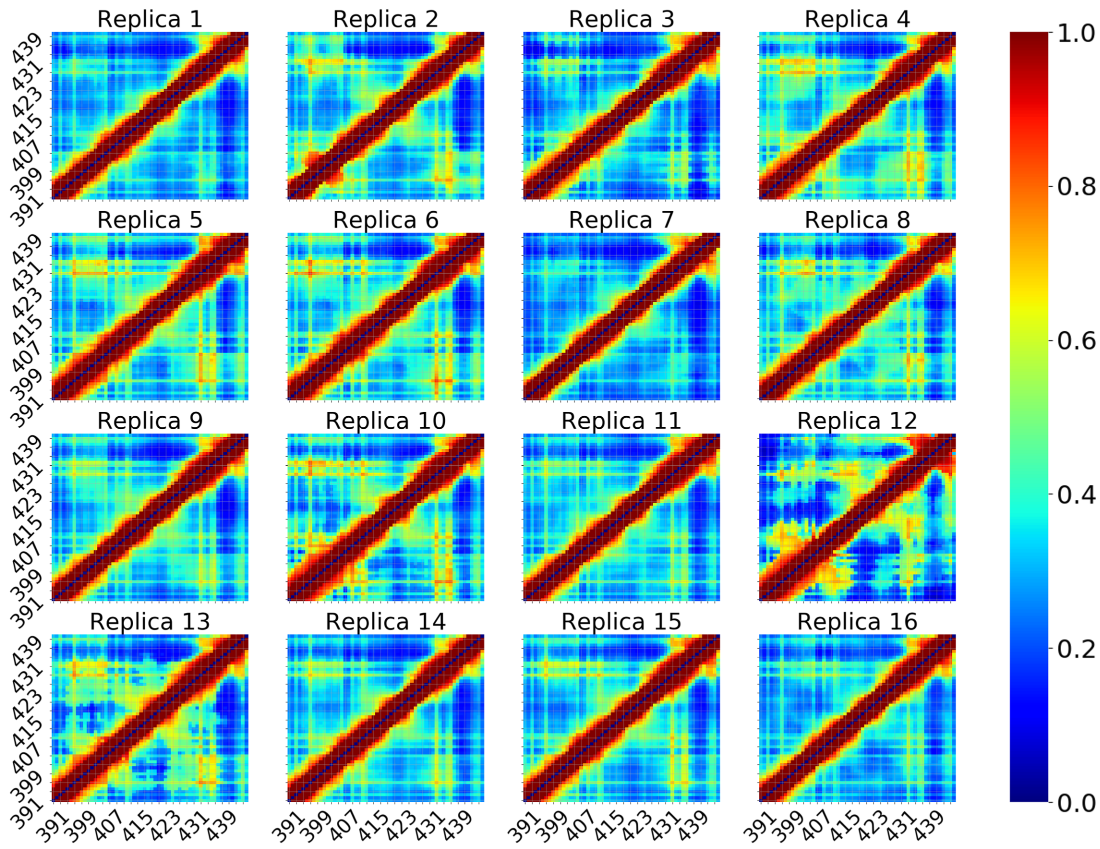

Supplementary Figure 6. Intramolecular contact maps of demultiplexed replicas from REST2 MD simulations of apo Tau-5$_{R2_R3}$. Comparison of the intramolecular contact probabilities observed in the 16 demultiplexed replicas of an apo Tau-5$_{R2_R3}$ REST2 MD simulation. Contacts between two residues were defined using a distance cutoff of 12Å between Cα atoms. Replica 7 shows substantially less populated contacts the R2 and R3 regions relative to the other replicas, and Replica 12 shows an elevated contact propensity between residues 390-395 and residues 400-405 within the R2 region.

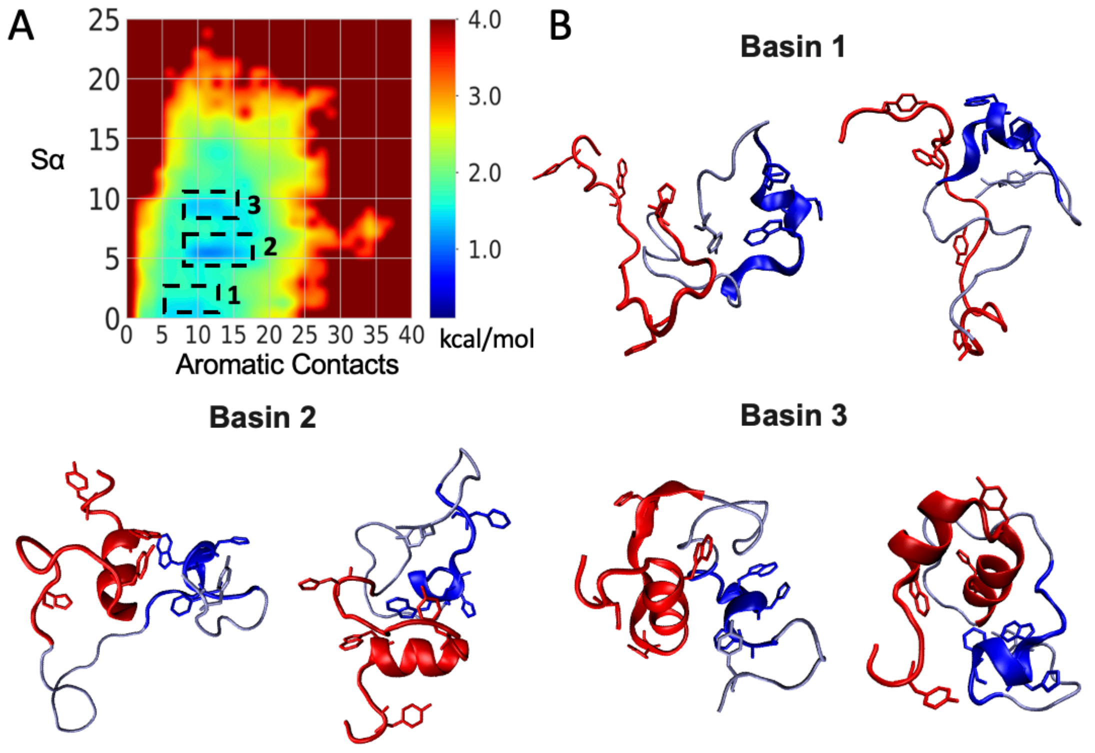

Supplementary Figure 7. Free energy surface of apo Tau-5$_{R2_R3}$ as a function aromatic contacts and helical content. A) Free energy surface of apo Tau-5$_{R2_R3}$ observed in the 300K replica of a 74µs explicit solvent REST2 MD simulation run with the a99SB-*disp* force field as function of the α-helical Sα and the number of contacts observed between aromatic residues. B) Representative snapshots of Tau-5$_{R2_R3}$ selected from the three most populated free energy basins in panel A. We define aromatic between two aromatic residues in Tau-5$_{R2_R3}$ as occurring in any frame where at least one heavy (non-hydrogen) atom of one residue is found within 4.0Å of a heavy atom of the second residue. For each aromatic contact when the minimum heavy atom distance is < 4.0Å, it will contribute a value of 1 to total number of aromatic contacts. When distance is > 7.0Å, it contributes a value 0 to the total number of aromatic contacts. For intermediate distances, the switching function $\left(1 - \frac{1}{1+e^{-3x+16.3}}\right)$ was used to convert the distances to values between 1 and 0.

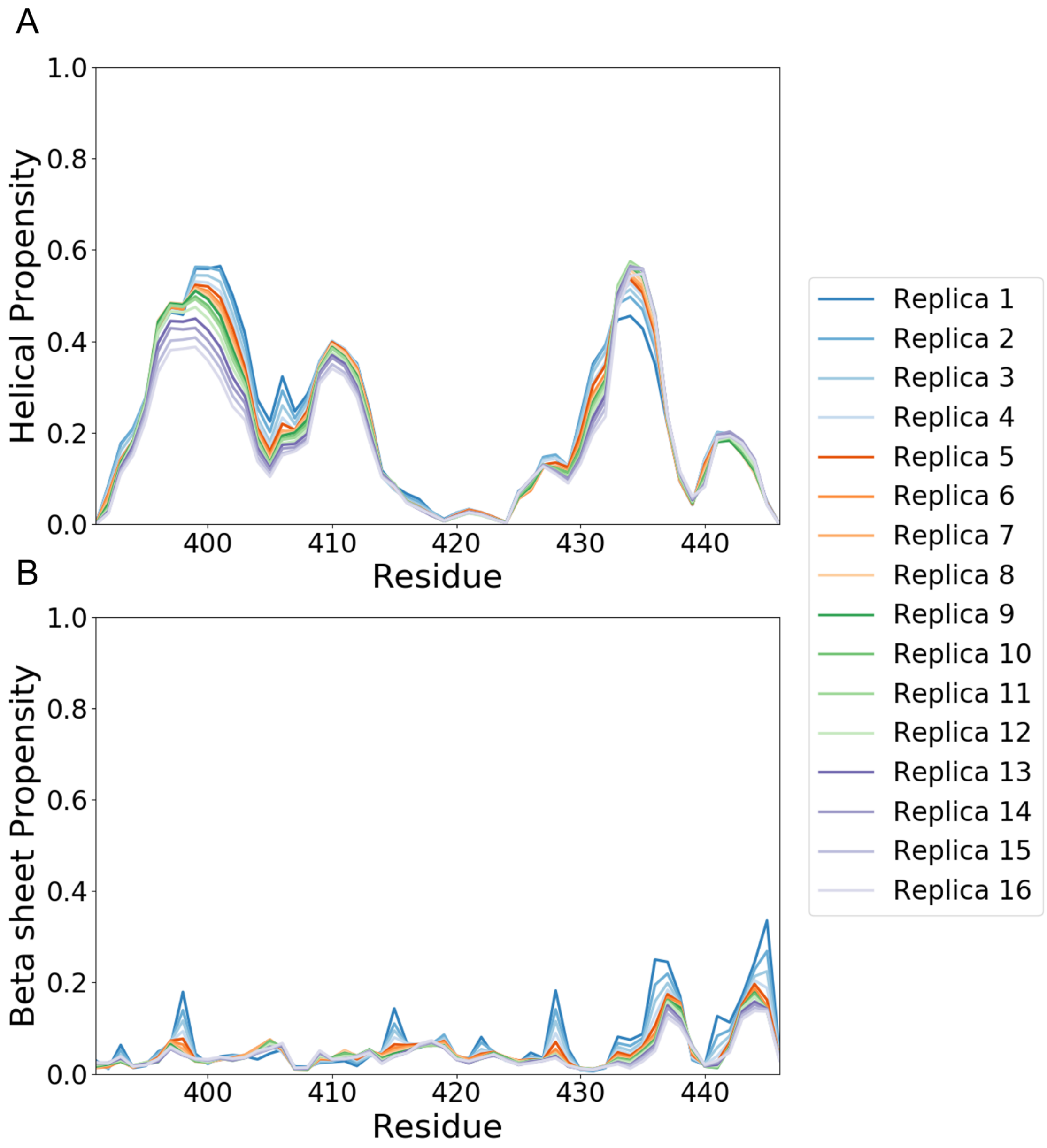

Supplementary Figure 8. Secondary structure propensities observed in REST2 MD simulations of Tau-5$_{R2_R3}$ in the presence of EPI-002. Comparison of α-helical (A) and β-sheet (B) propensities of Tau-5$_{R2_R3}$ observed in the 16 solute temperature rungs of a REST2 MD simulations of Tau-5$_{R2_R3}$ in the presence of EPI-002. Secondary structure content is calculated by the DSSP algorithm.

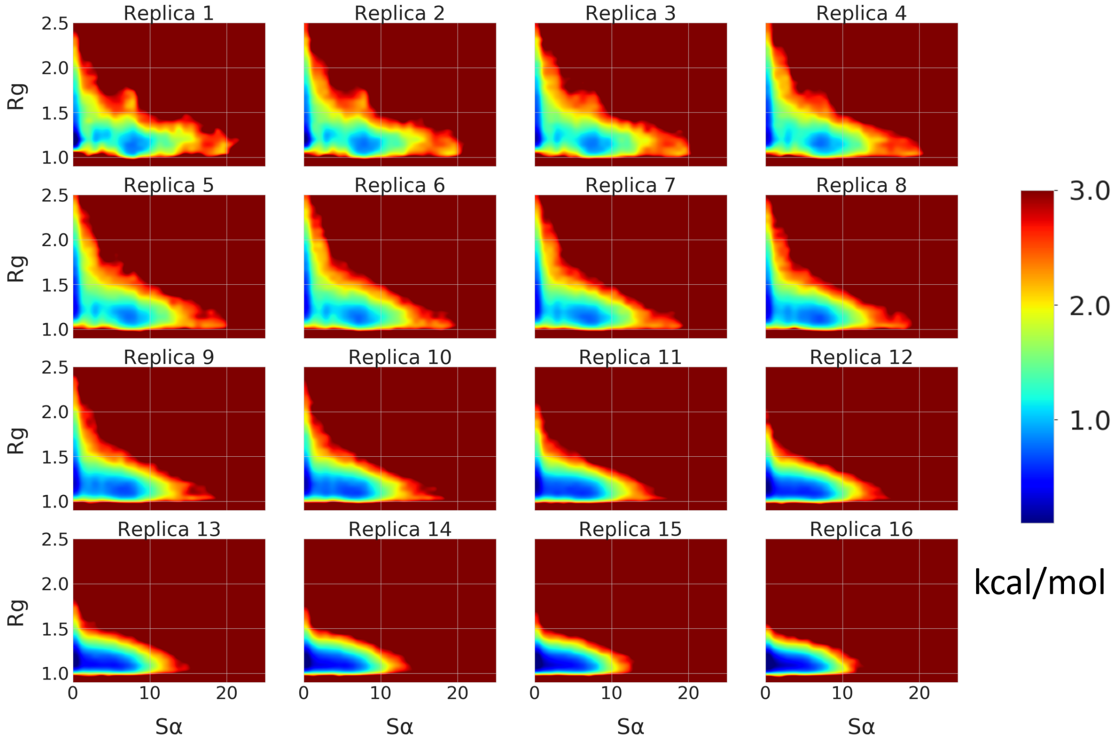

Supplementary Figure 9. Free energy surfaces from REST2 MD simulations of Tau-5$_{R2_R3}$ in the presence of EPI-002. Comparison of free energy surfaces of Tau-5$_{R2_R3}$ conformations as a function of the α-helical order parameter Sα and radius of gyration (R_g) for the 16 solute temperature rungs of a REST2 MD simulation of Tau-5$_{R2_R3}$ in the presence of EPI-002. R_g is reported in nm.

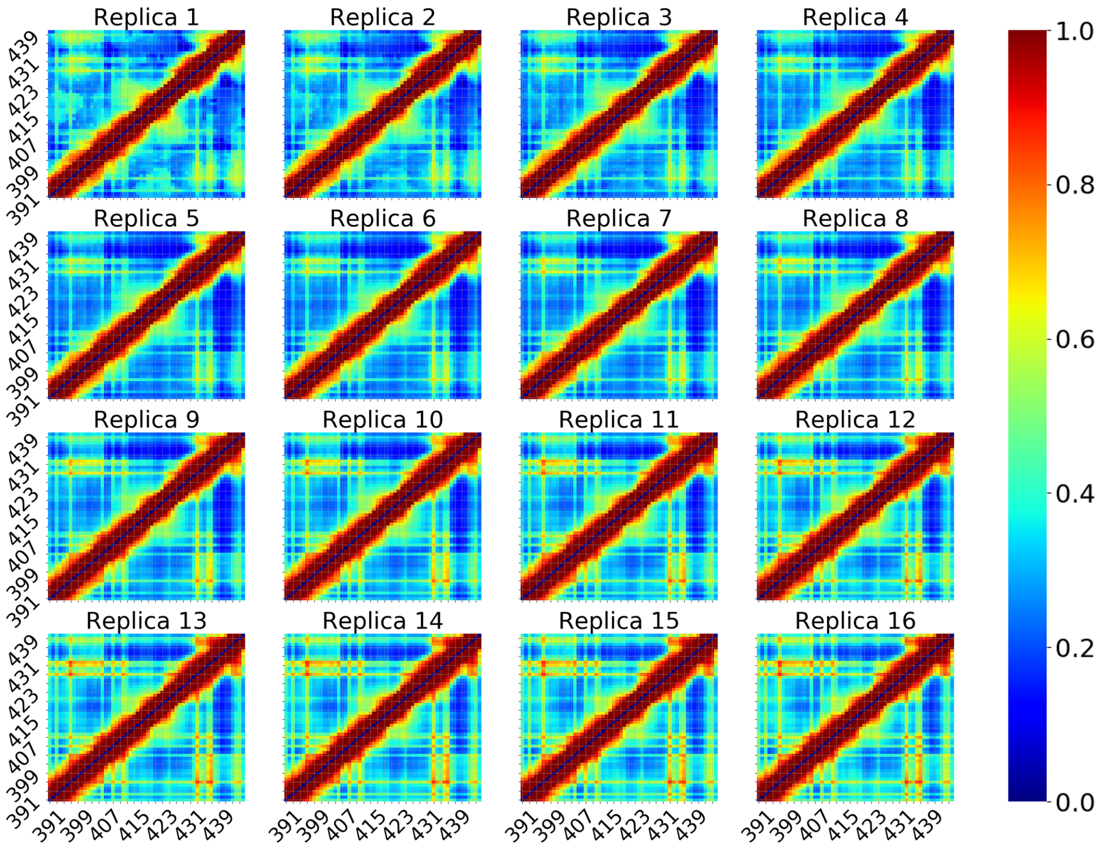

Supplementary Figure 10. Intramolecular contact maps from REST2 MD simulations of Tau-5$_{R2_R3}$ in the presence of EPI-002. Comparison of the intramolecular contact probabilities observed in the 16 solute temperature rungs of a REST2 MD simulation of Tau-5$_{R2_R3}$ in the presence of EPI-002. Contacts between two residues were defined using a distance cutoff of 12Å between Cα atoms.

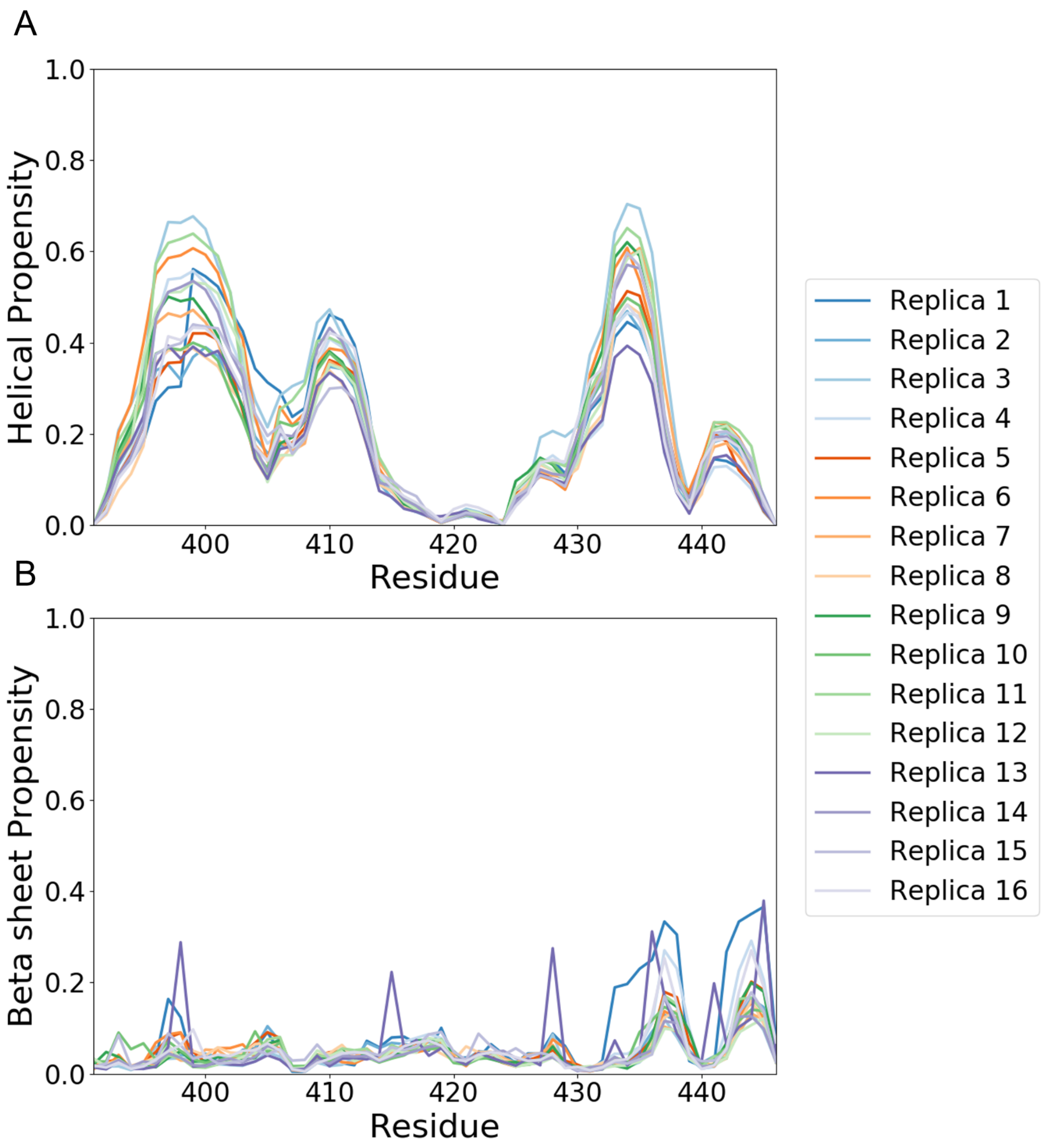

Supplementary Figure 11. Secondary structure propensities observed in demultiplexed replicas from REST2 MD simulations of Tau-5$_{R2_R3}$ in the presence of EPI-002. Comparison of α-helical (A) and β-sheet (B) propensities of Tau-5$_{R2_R3}$ observed in the 16 demultiplexed replicas of a REST2 MD simulations of Tau-5$_{R2_R3}$ in the presence of EPI-002. Secondary structure content is calculated by the DSSP algorithm.

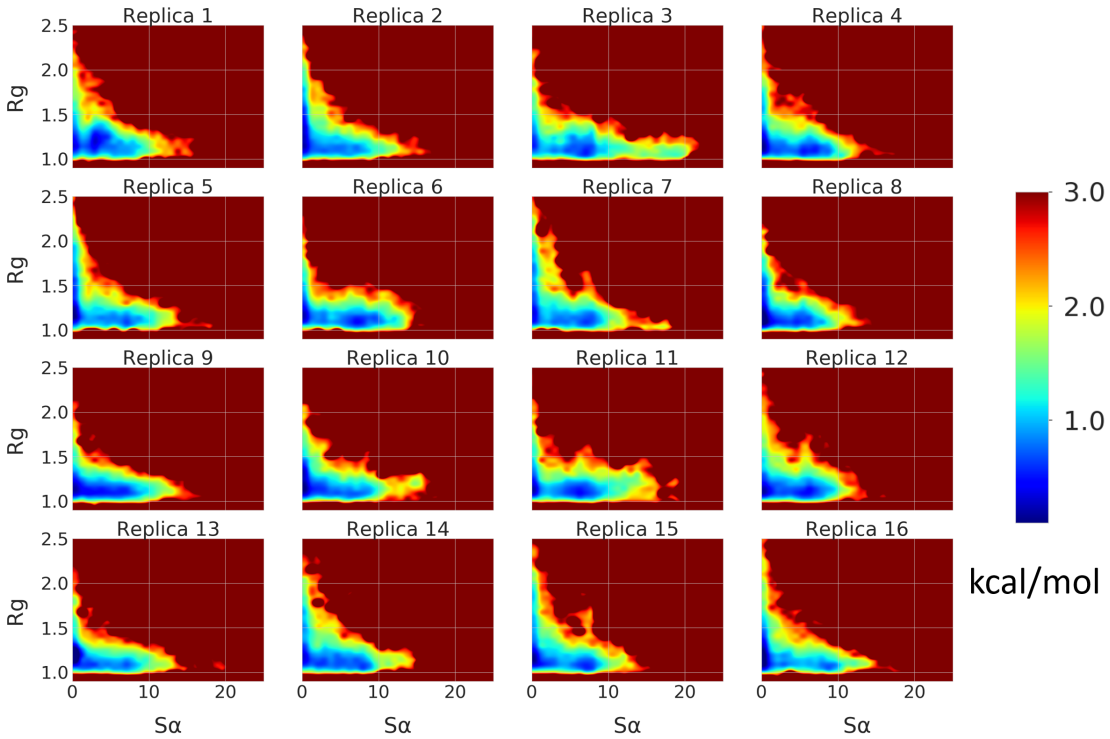

Supplementary Figure 12. Free energy surfaces of demultiplexed replicas from REST2 MD simulations of Tau-5$_{R2_R3}$ in the presence of EPI-002. Comparison of free energy surfaces of Tau-5$_{R2_R3}$ conformations as a function of the α-helical order parameter Sα and radius of gyration (R_g) for the 16 demultiplexed replicas of a REST2 MD simulations of Tau-5$_{R2_R3}$ in the presence of EPI-002. R_g is reported in nm.

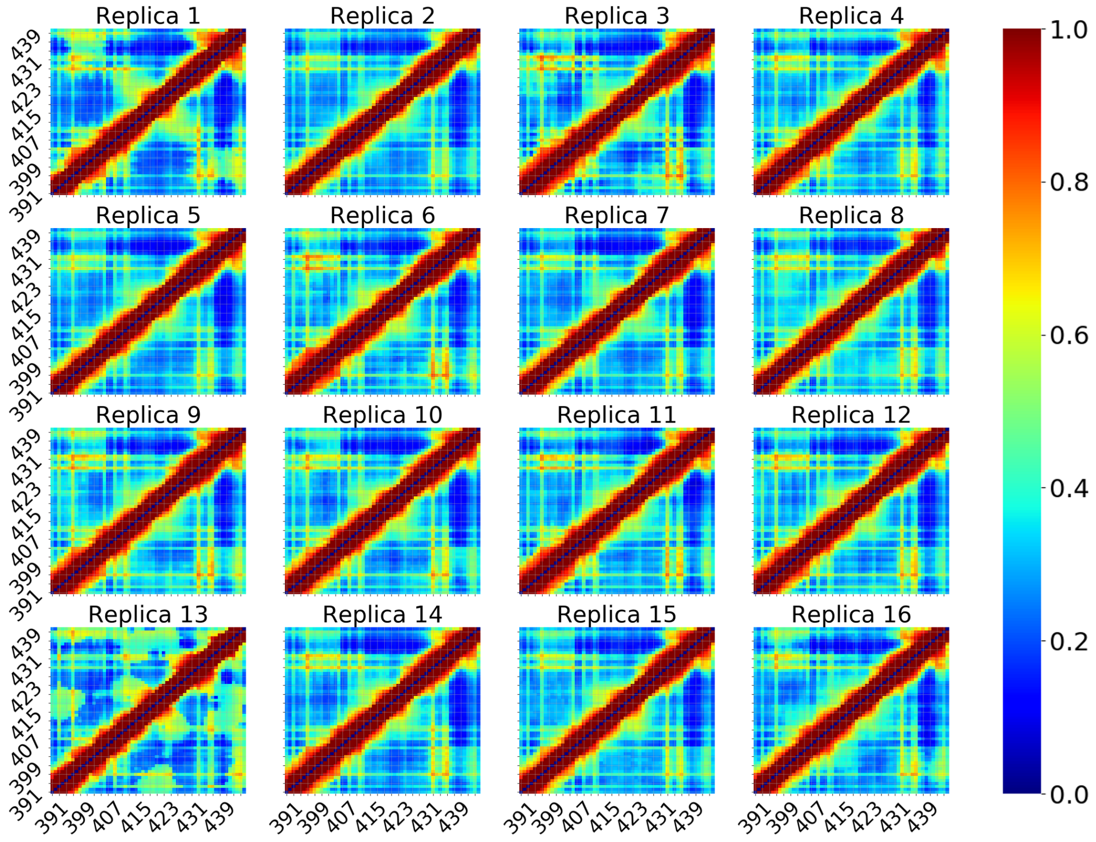

Supplementary Figure 13. Intramolecular contact maps of demultiplexed replicas from REST2 MD simulations of Tau-5$_{R2_R3}$ in the presence of EPI-002. Comparison of the intramolecular contact probabilities observed in the 16 demultiplexed replicas of a REST2 MD simulations of Tau-5$_{R2_R3}$ in the presence of EPI-002. Contacts between two residues were defined using a distance cutoff of 12Å between Cα atoms.

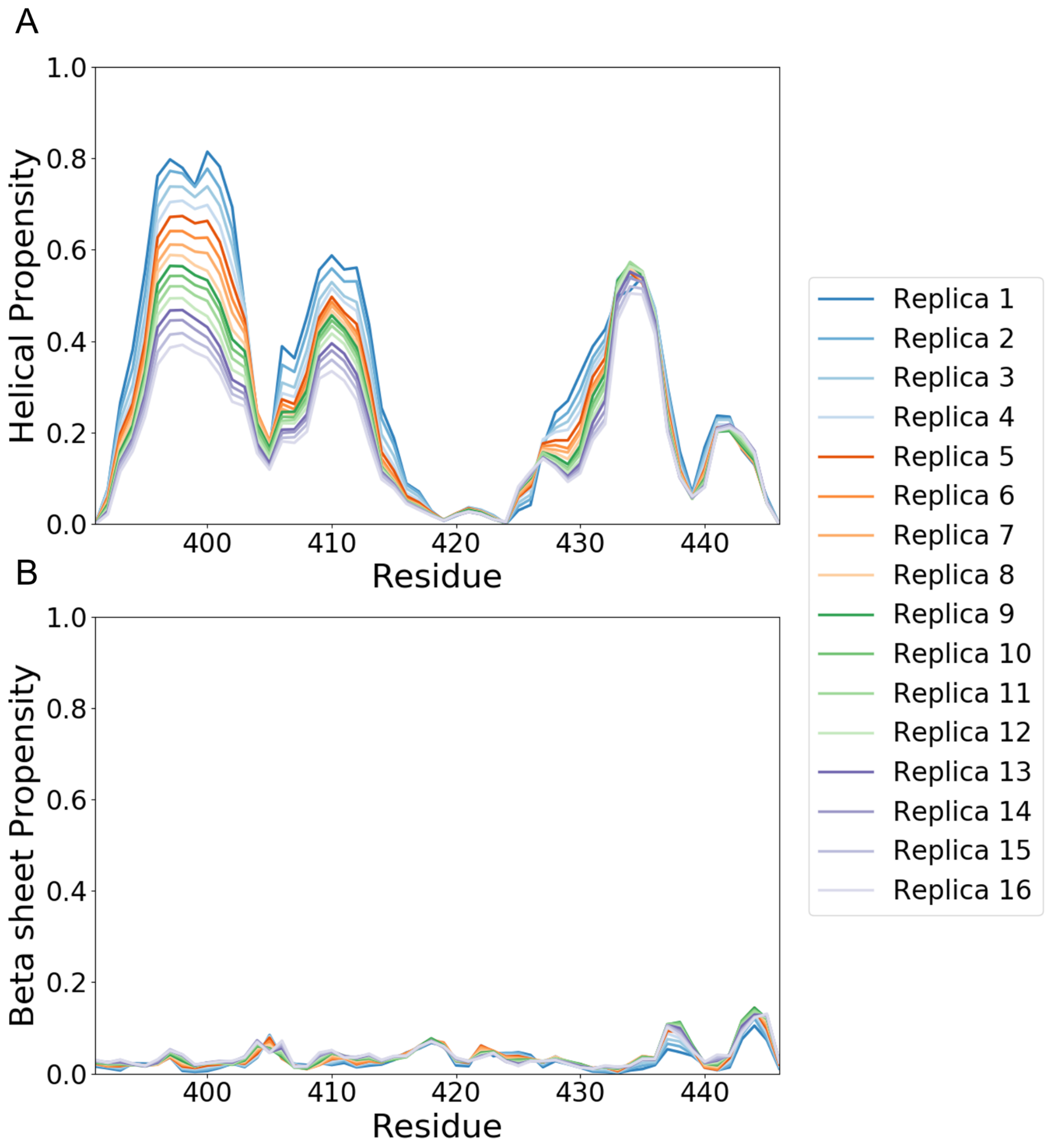

Supplementary Figure 14. Secondary structure propensities observed in REST2 MD simulations of Tau-5$_{R2_R3}$ in the presence of EPI-7170. Comparison of α-helical (A) and β-sheet (B) propensities of Tau-5$_{R2_R3}$ observed in the 16 solute temperature rungs of a REST2 MD simulations of Tau-5$_{R2_R3}$ in the presence of EPI-7170. Secondary structure content is calculated by the DSSP algorithm.

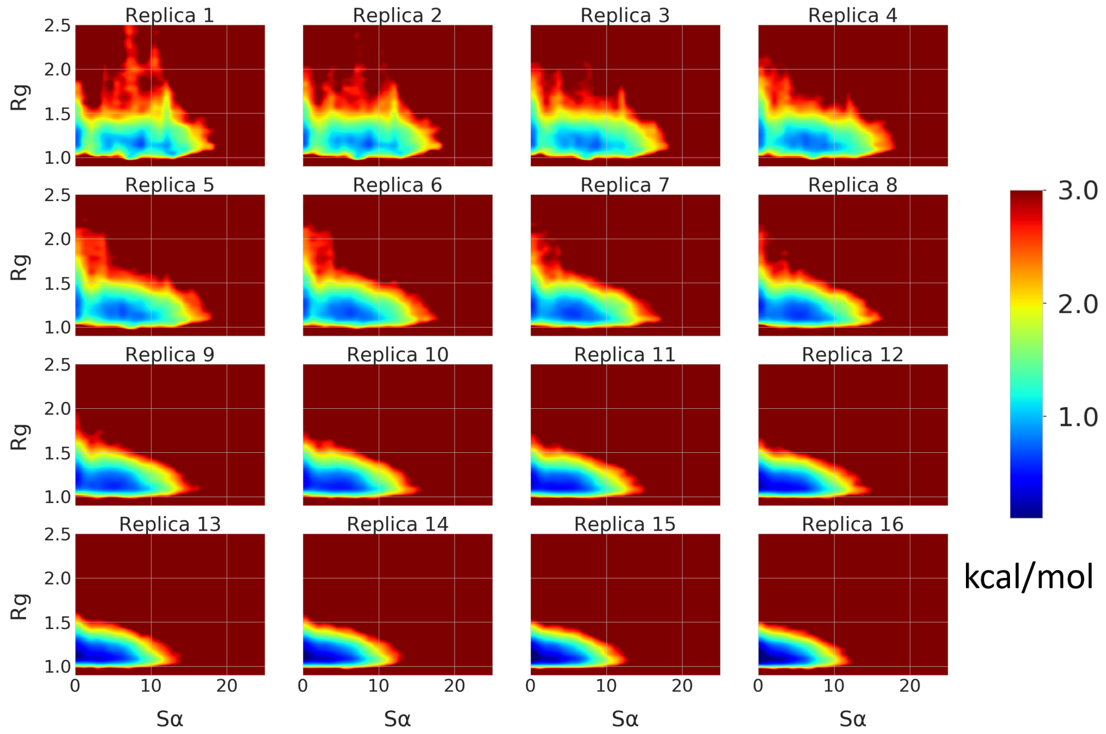

Supplementary Figure 15. **Free energy surfaces from REST2 MD simulations of Tau-5$_{R2_R3}$ in the presence of EPI-7170.** Comparison of free energy surfaces of Tau-5$_{R2_R3}$ conformations as a function of the α-helical order parameter Sα and radius of gyration (R_g) for the 16 solute temperature rungs of a REST2 MD simulation of Tau-5$_{R2_R3}$ in the presence of EPI-7170.

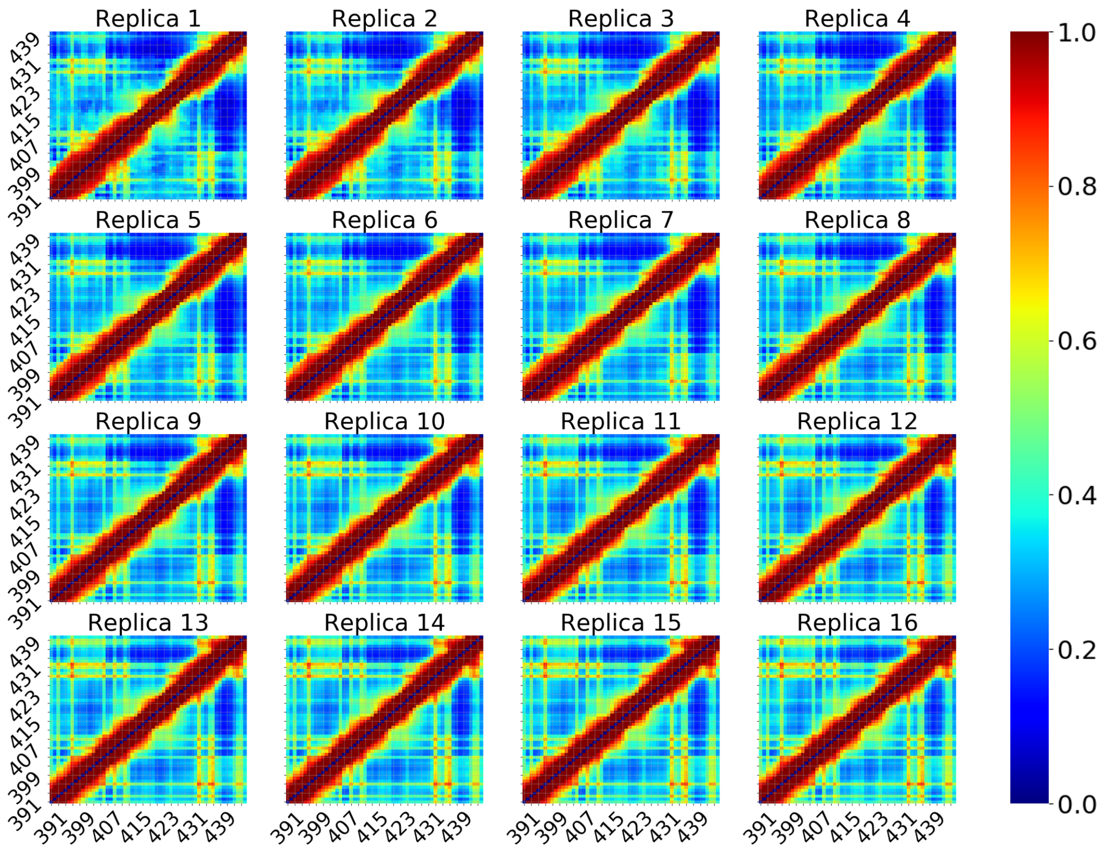

Supplementary Figure 16. **Intramolecular contact maps from REST2 MD simulations of Tau-5$_{R2_R3}$ in the presence of EPI-7170.** Comparison of the intramolecular contact probabilities observed in the 16 solute temperature rungs of a REST2 MD simulation of Tau-5$_{R2_R3}$ in the presence of EPI-7170. Contacts between two residues were defined as occurring using a distance cutoff of 12Å between Cα atoms.

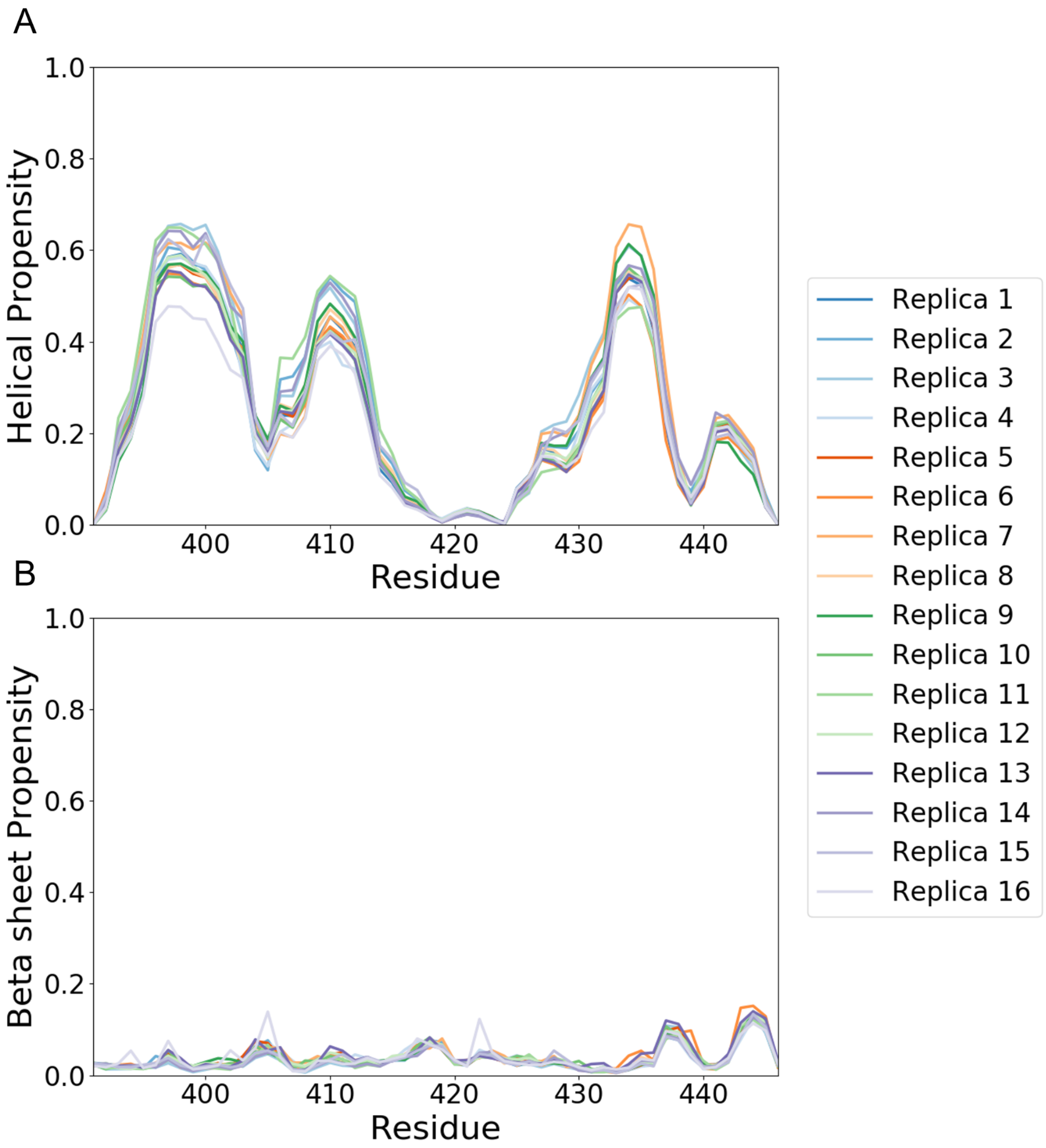

Supplementary Figure 17. Secondary structure propensities observed in demultiplexed replicas from REST2 MD simulations of Tau-5$_{R2_R3}$ in the presence of EPI-7170. Comparison of α-helical (A) and β-sheet (B) propensities of Tau-5$_{R2_R3}$ observed in the 16 demultiplexed replicas of a REST2 MD simulations of Tau-5$_{R2_R3}$ in the presence of EPI-7170. Secondary structure content is calculated by the DSSP algorithm.

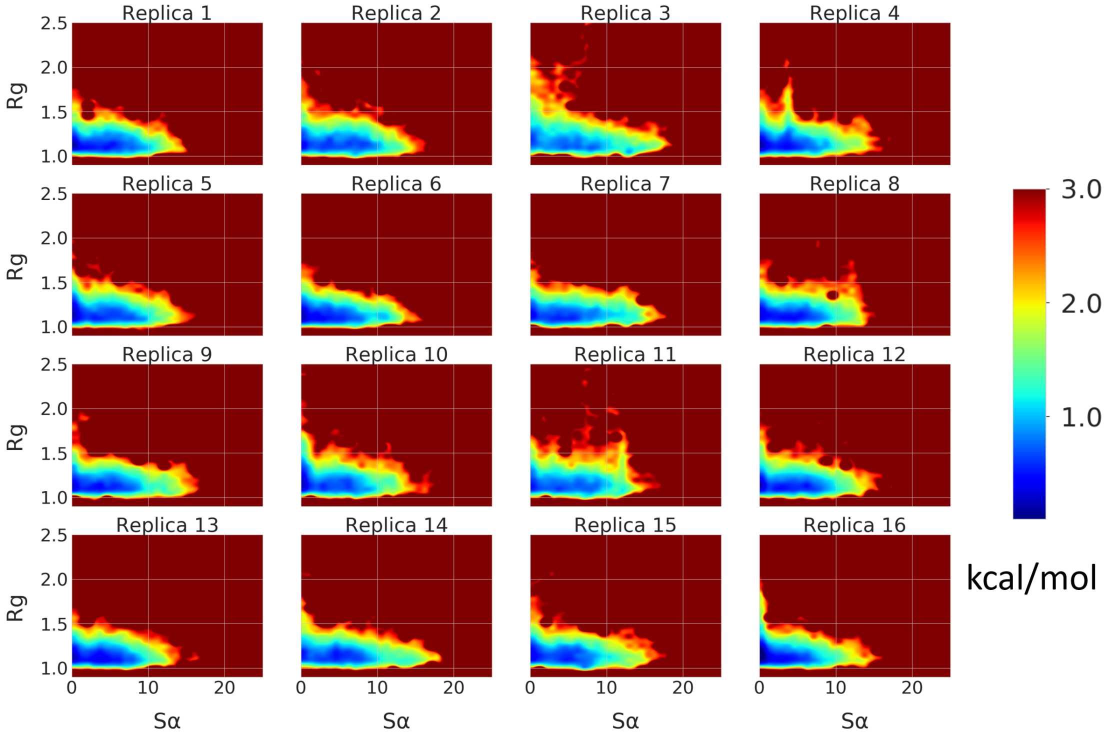

Supplementary Figure 18. Free energy surfaces of demultiplexed replicas from REST2 MD simulations of Tau-5$_{R2_R3}$ in the presence of EPI-7170. Comparison of free energy surfaces of Tau-5$_{R2_R3}$ conformations as a function of the α-helical order parameter Sα and radius of gyration (R_g) for the 16 demultiplexed replicas of a REST2 MD simulations of Tau-5$_{R2_R3}$ in the presence of EPI-7170. R_g is reported in nm.

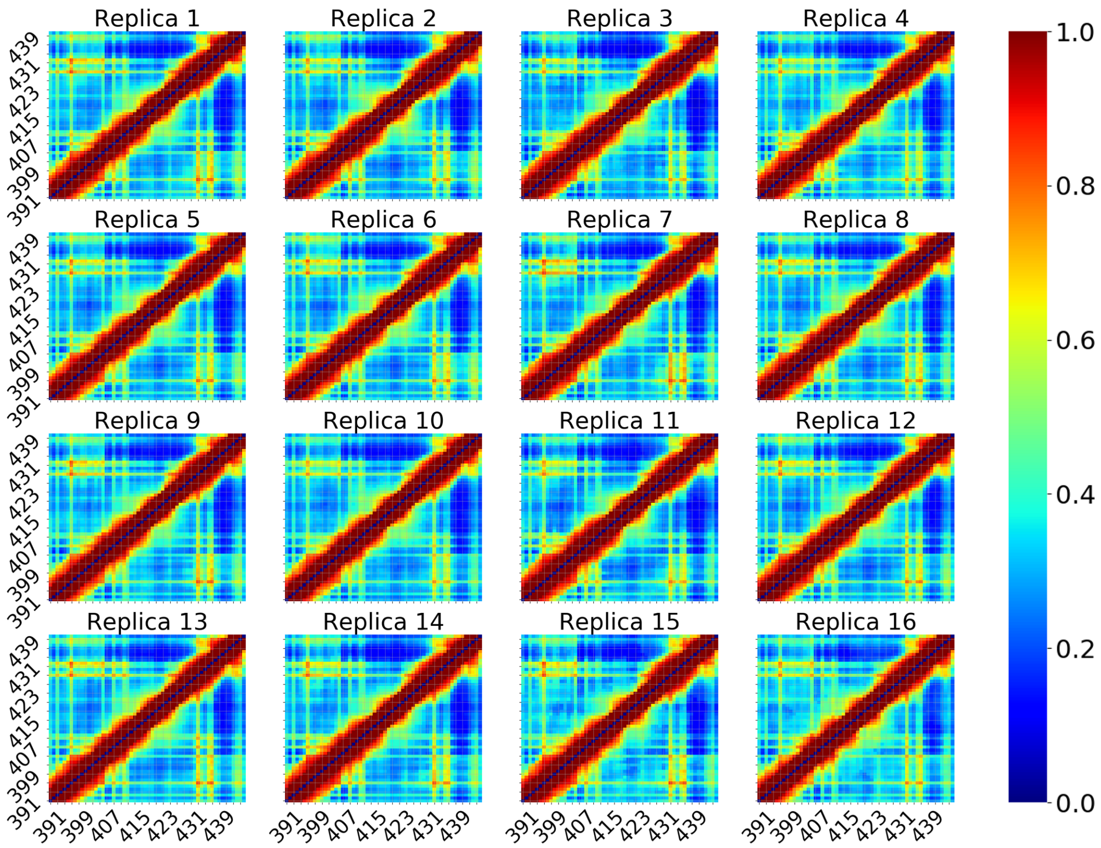

Supplementary Figure 19. Intramolecular contact maps of demultiplexed replicas from REST2 MD simulations of Tau-5$_{R2_R3}$ in the presence of EPI-7170. Comparison of the intramolecular contact probabilities observed in the 16 demultiplexed replicas of a REST2 MD simulations of Tau-5$_{R2_R3}$ in the presence of EPI-7170. Contacts between two residues were defined using a distance cutoff of 12Å between Cα atoms.

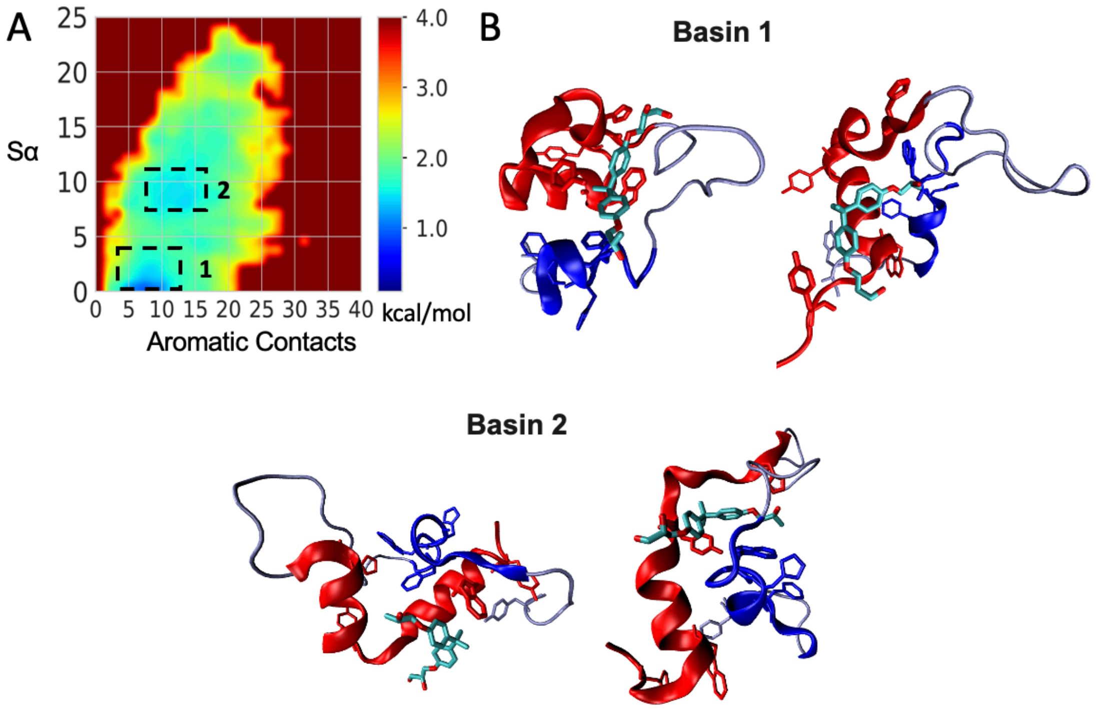

Supplementary Figure 20. **Free energy surface of Tau-5$_{R2_R3}$ in the presence of EPI-002 as a function aromatic contacts and helical content.** A) Free energy surface of Tau-5$_{R2_R3}$ observed in the 300K replica of an explicit solvent REST2 MD simulation of Tau-5$_{R2_R3}$ in the presence of EPI-002 run with the a99SB-*disp* and GAFF1 force fields as function of Sα and the number of contacts observed between aromatic residues in Tau-5$_{R2_R3}$ and EPI-002 and aromatic residues in Tau-5$_{R2_R3}$. B) Representative snapshots of Tau-5$_{R2_R3}$ and EPI-002 selected from the two most populated free energy basins in panel A. We define aromatic contacts between EPI-002 and an aromatic residue, or between two aromatic residues in Tau-5$_{R2_R3}$ as occurring in any frame where at least one heavy (non-hydrogen) atom of one residue is found within 4.0Å of a heavy atom of the second residue. For each aromatic contact when the minimum heavy atom distance is < 4.0Å, it will contribute a value of 1 to total number of aromatic contacts. When distance is > 7.0Å, it contributes a value 0 to the total number of aromatic contacts. For intermediate distances, the switching function $\left(1 - \frac{1}{1+e^{-3x+16.3}}\right)$ was used to convert the distances to values between 1 and 0.

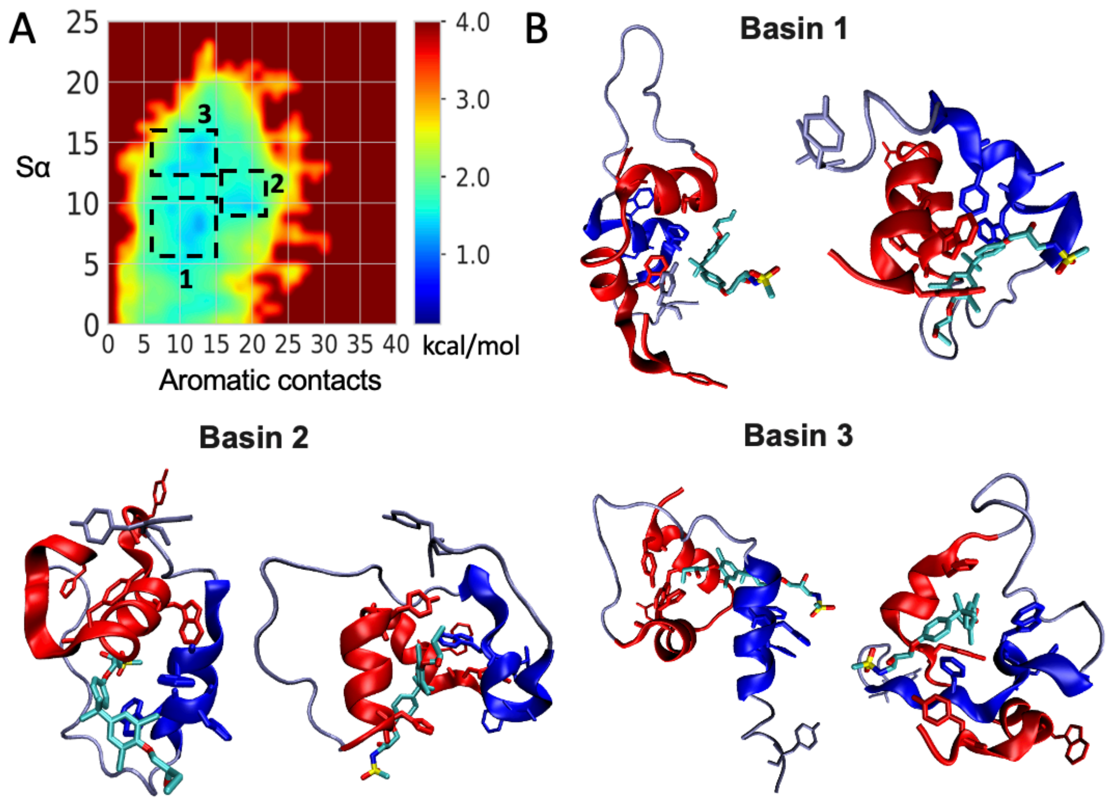

Supplementary Figure 21. Free energy surface of Tau-5$_{R2_R3}$ in the presence of EPI-002 as a function aromatic contacts and helical content. A) Free energy surface of Tau-5$_{R2_R3}$ observed in the 300K replica of an explicit solvent REST2 MD simulation of Tau-5$_{R2_R3}$ in the presence of EPI-7170 run with the a99SB-*disp* and GAFF1 force fields as function of Sα and the number of contacts observed between aromatic residues in Tau-5$_{R2_R3}$ and number of contacts between EPI-7170 and aromatic residues in Tau-5$_{R2_R3}$. B) Representative snapshots of Tau-5$_{R2_R3}$ and EPI-7170 selected from the the three most populated free energy basins in panel A.

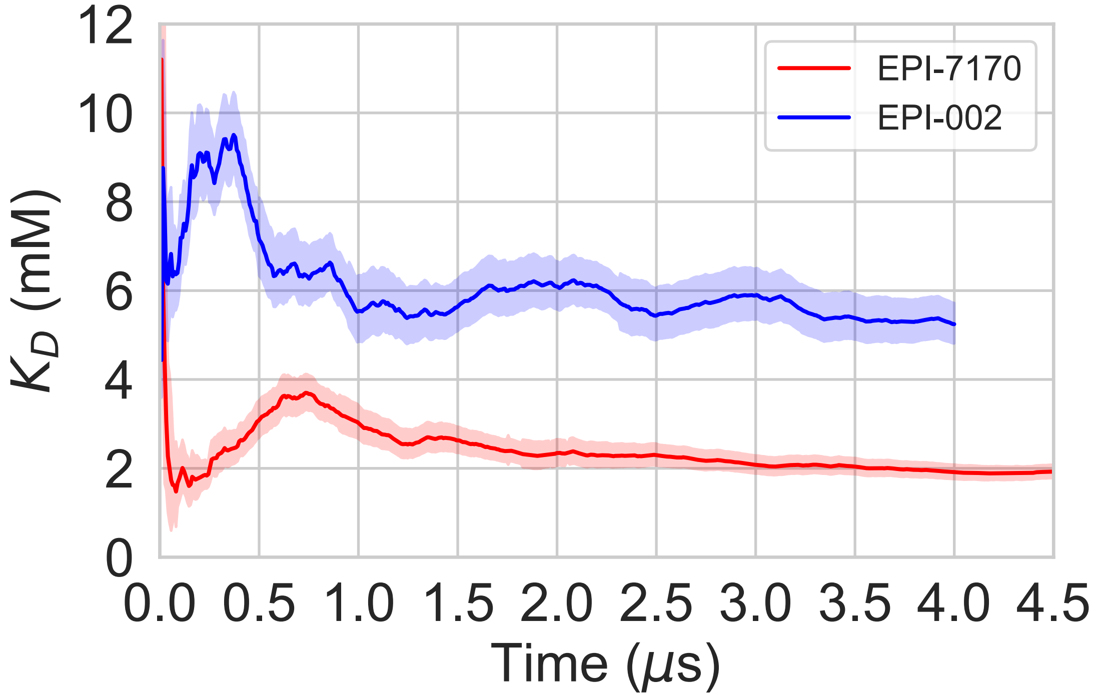

Supplementary Figure 22. Convergence of calculated K_D values of EPI-002 and EPI-7170. Convergence of calculated K_D values for simulations of Tau-5_{R2_R3} in the presence of EPI-002 (Blue) and EPI-7170 (Red). The K_D values for each time point are reported as the mean value of the K_D observed in all frames of the trajectory prior to that time point +/- statistical error estimates (shaded regions). Statistical error estimates were calculated from a blocking analysis using all frames in the trajectory prior to that time point.

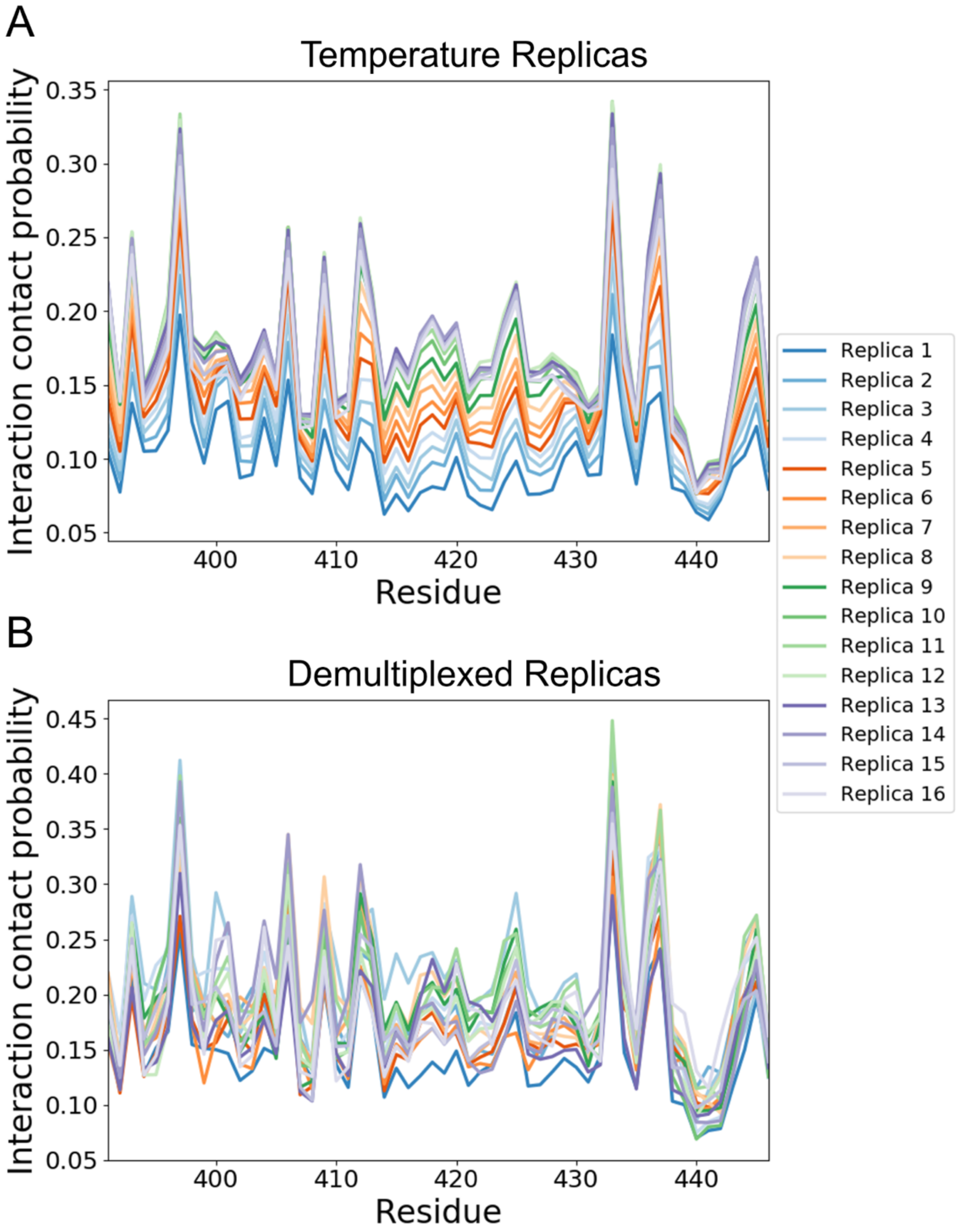

Supplementary Figure 23. Per-residue ligand-protein contact probabilities observed in REST2 MD simulations of Tau-5$_{R2_R3}$ in the presence of EPI-002. A) Ligand:protein intermolecular contact probabilities observed in the 16 solute temperature rungs from a REST2 MD simulation of Tau-5$_{R2_R3}$ in the presence of EPI-002. Contacts between are defined between EPI-002 and Tau-5$_{R2_R3}$ residues using a cutoff of 6Å between heavy atoms. B) Ligand:protein intermolecular contact probabilities observed in the 16 demultiplexed replicas.

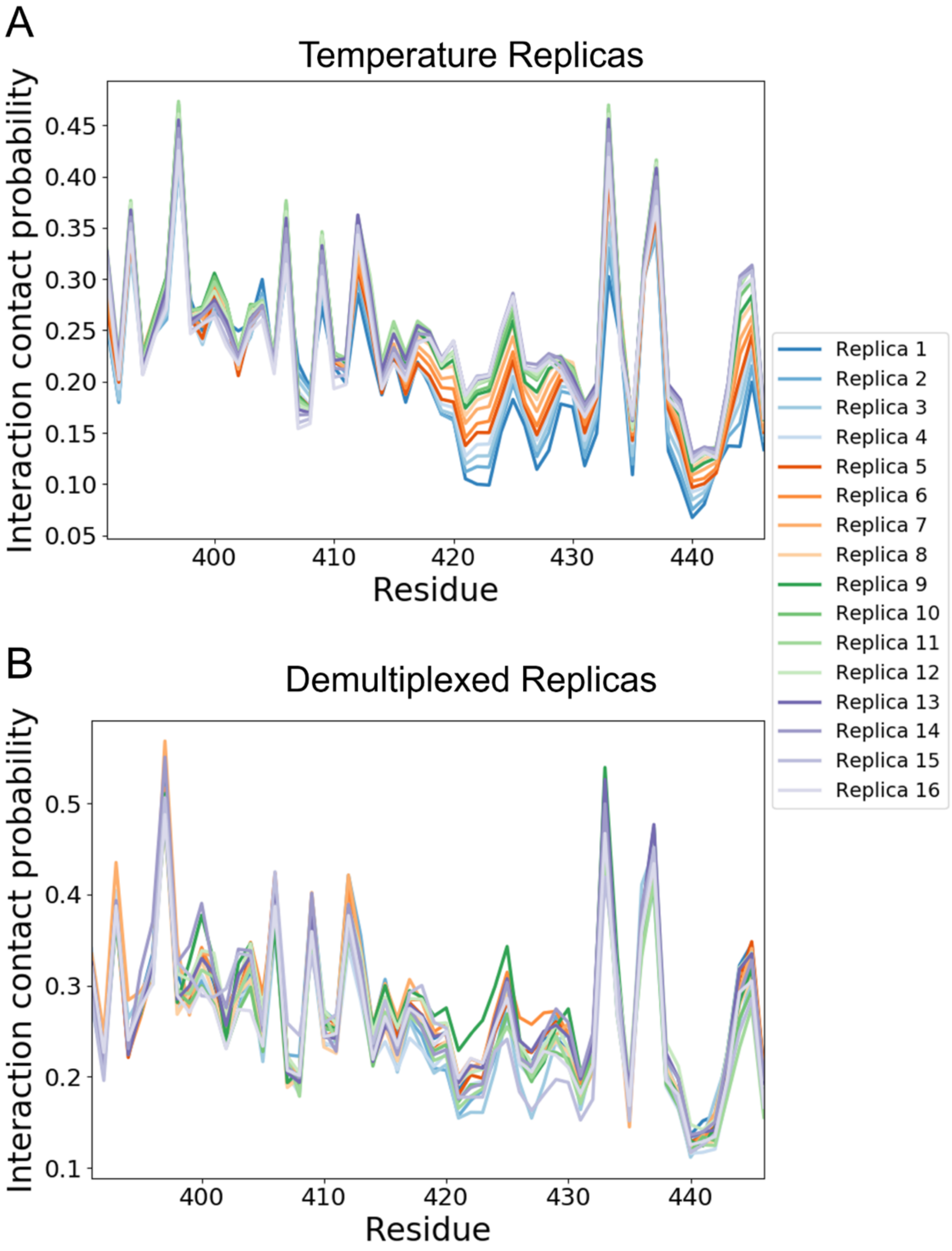

Supplementary Figure 24. Per-residue ligand-protein contact probabilities observed in REST2 MD simulations of Tau-5$_{R2_R3}$ in the presence of EPI-7170. A) Ligand:protein intermolecular contact probabilities observed in the 16 solute temperature rungs from a REST2 MD simulation of Tau-5$_{R2_R3}$ in the presence of EPI-7170. Contacts between are defined between EPI-7170 and Tau-5$_{R2_R3}$ residues using a cutoff of 6Å between closest heavy atoms. B) Ligand:protein intermolecular contact probabilities observed in the 16 demultiplexed replicas.

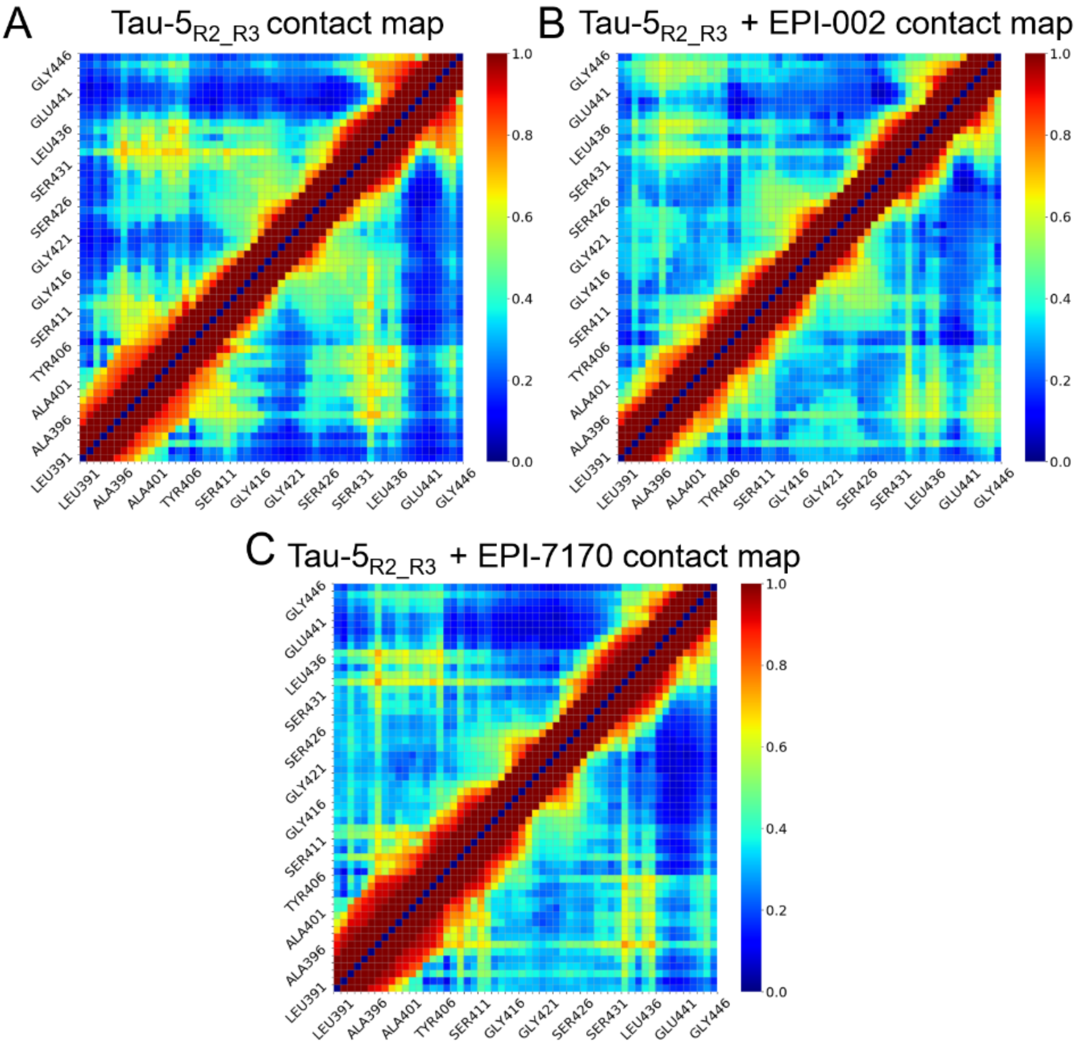

Supplementary Figure 25. Intramolecular contact maps of Tau-5$_{R2_R3}$ observed in REST2 MD simulations in the presence and absence of ligands. Comparison of intramolecular contact probabilities of the 300K solute temperature replicas of REST2 MD simulations of apo Tau-5$_{R2_R3}$ (A), the EPI-002:Tau-5$_{R2_R3}$ bound ensemble (B) and the EPI-7170:Tau-5$_{R2_R3}$ bound ensemble (C). Contacts between residues are defined using a cutoff distance of 12Å between Cα atoms.

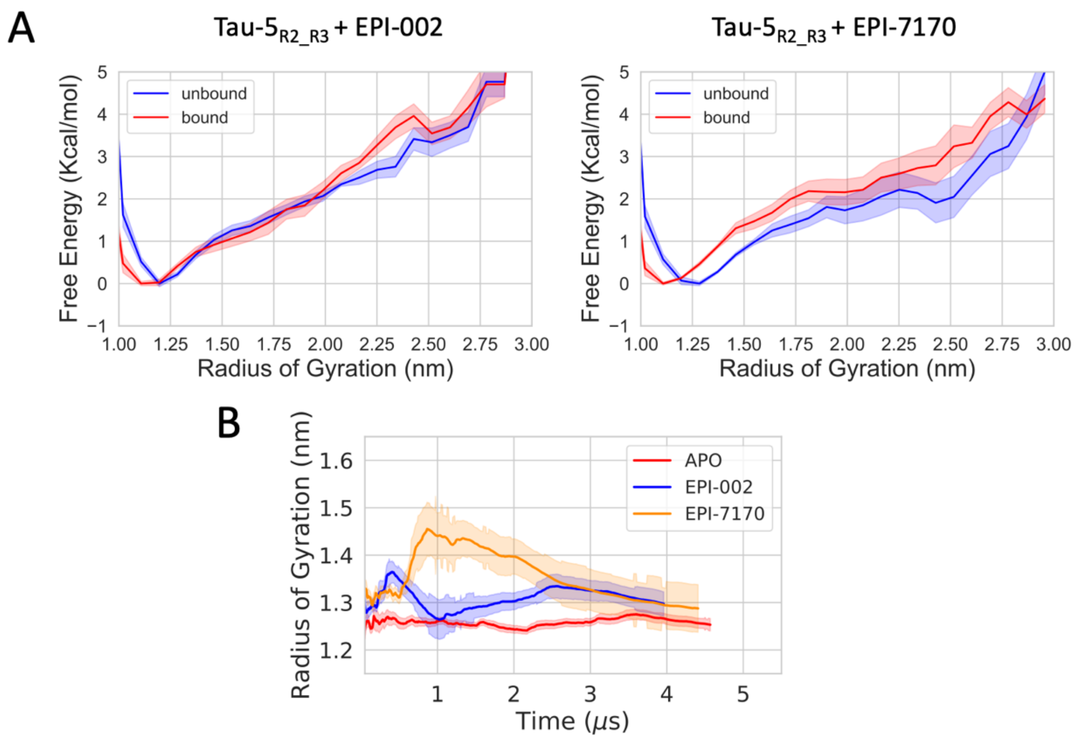

Supplementary Figure 26. Radius of gyration of Tau-5$_{R2_R3}$ observed in REST2 MD simulations in the presence and absence of ligands. A) Comparison of the Radius of Gyration (R_g) in bound and unbound states from REST2 MD simulations of Tau-5$_{R2_R3}$ in the presence of EPI-002 and Tau-5$_{R2_R3}$ in the presence of EPI-7170. Bound states are defined as all frames containing at least 1 intermolecular contact (heavy atom pairs within 6Å) between a ligand and Tau-5$_{R2_R3}$. Free energies were calculated using the R_g values of all bound or unbound frames of each MD trajectories, and shaded regions indicate the standard error of the calculated free energies when the trajectory is split into 5 equally sized blocks. B) Convergence of calculated Tau-5$_{R2_R3}$ R_g values for simulations of apo Tau-5$_{R2_R3}$ (red) and Tau-5$_{R2_R3}$ in the presence of EPI-002 (nlue) and EPI-7170 (orange). R_g values for each time point are reported as the mean value of the R_g observed in all frames of the trajectory prior to that time point +/- statistical error estimates (shaded regions). Statistical error estimates were calculated from a blocking analysis using all frames in the trajectory prior to that time point.

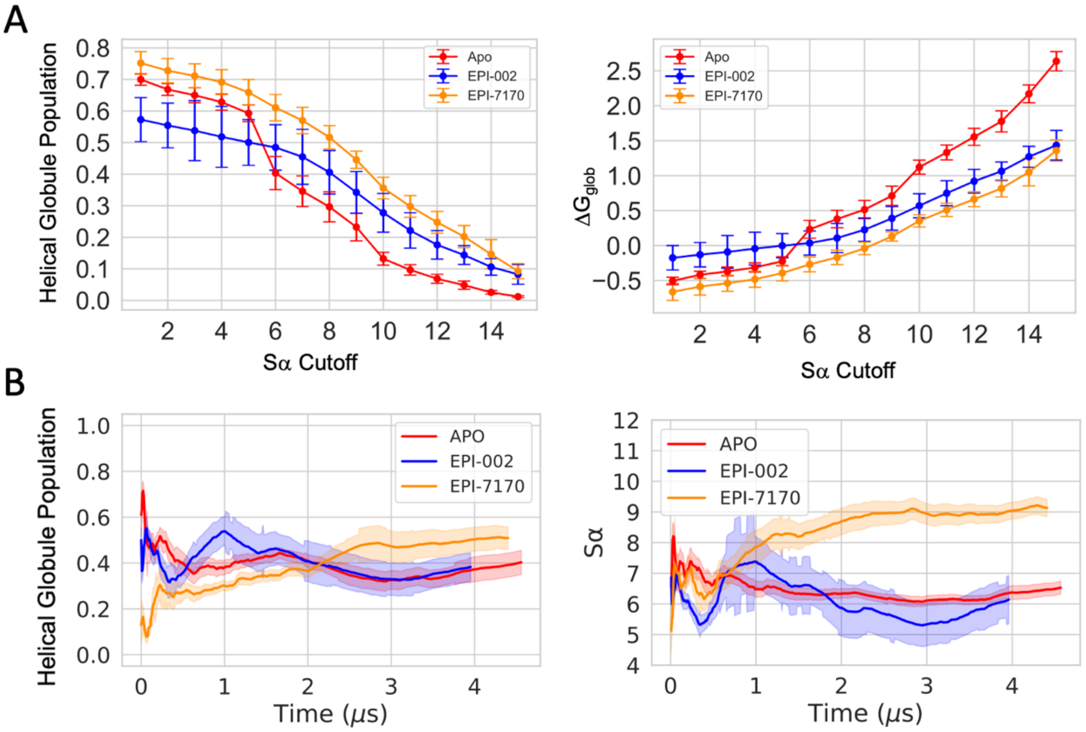

Supplementary Figure 27. Helical globule populations of Tau-5$_{R2_R3}$ observed in REST2 MD simulations in the presence and absence of ligands. A) Helical globule population and stability (ΔG_{glob}) relative to non-globule states in MD simulations of Tau-5$_{R2_R3}$ in its apo form and bound to EPI-002 and EPI-7170 as a function of the Sα cutoff used in the definition of the helical globule state. Helical globule states are defined as all conformations with R_g<1.3nm and Sα values greater than Sα cutoff**.** Data are reported as mean values +/- statistical error estimates obtained from blocking. B) Convergence of calculated Tau-5$_{R2_R3}$ Sα and helical globule population values for simulations of apo Tau-5$_{R2_R3}$ (red) and Tau-5$_{R2_R3}$ in the presence of EPI-002 (nlue) and EPI-7170 (orange). Values for each time point are reported as the mean value observed in all frames of the trajectory prior to that time point +/- statistical error estimates (shaded regions). Statistical error estimates were calculated from a blocking analysis using all frames in the trajectory prior to that time point.

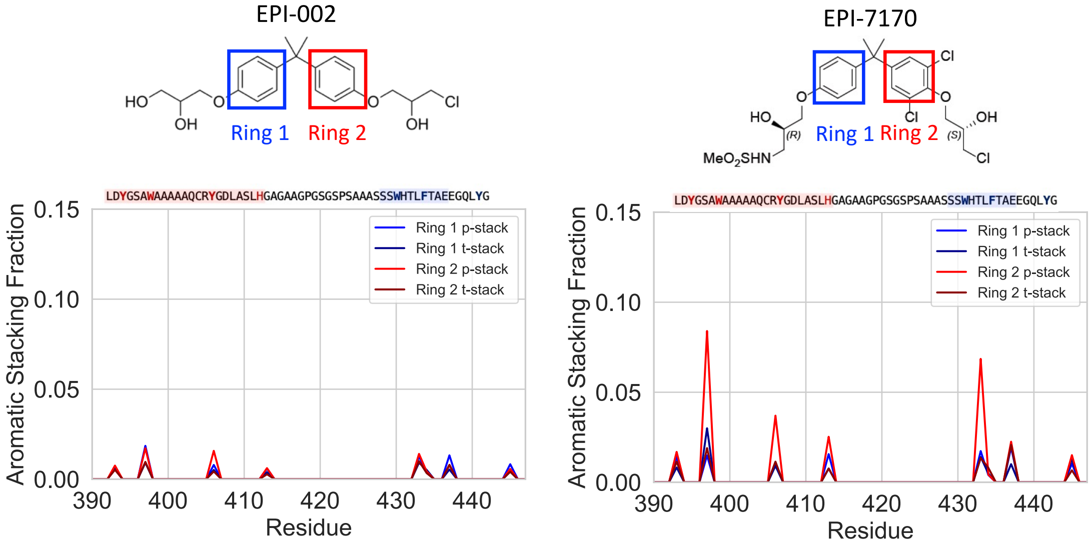

Supplementary Figure 28. Protein-ligand aromatic stacking interactions observed in the Tau-5$_{R2_R3}$:EPI-002 and Tau-5$_{R2_R3}$:EPI-7170 bound ensembles. Comparison of parallel stacking (p-stack) and t-stacking (t-stack) populations in the EPI-002:Tau-5$_{R2_R3}$ and EPI-7170:Tau-5$_{R2_R3}$ bound ensembles. The dichlorinated phenyl ring of EPI-7170 has a substantially larger population of parallel stacked conformations than the corresponding non-chlorinated phenyl ring in EPI-002.

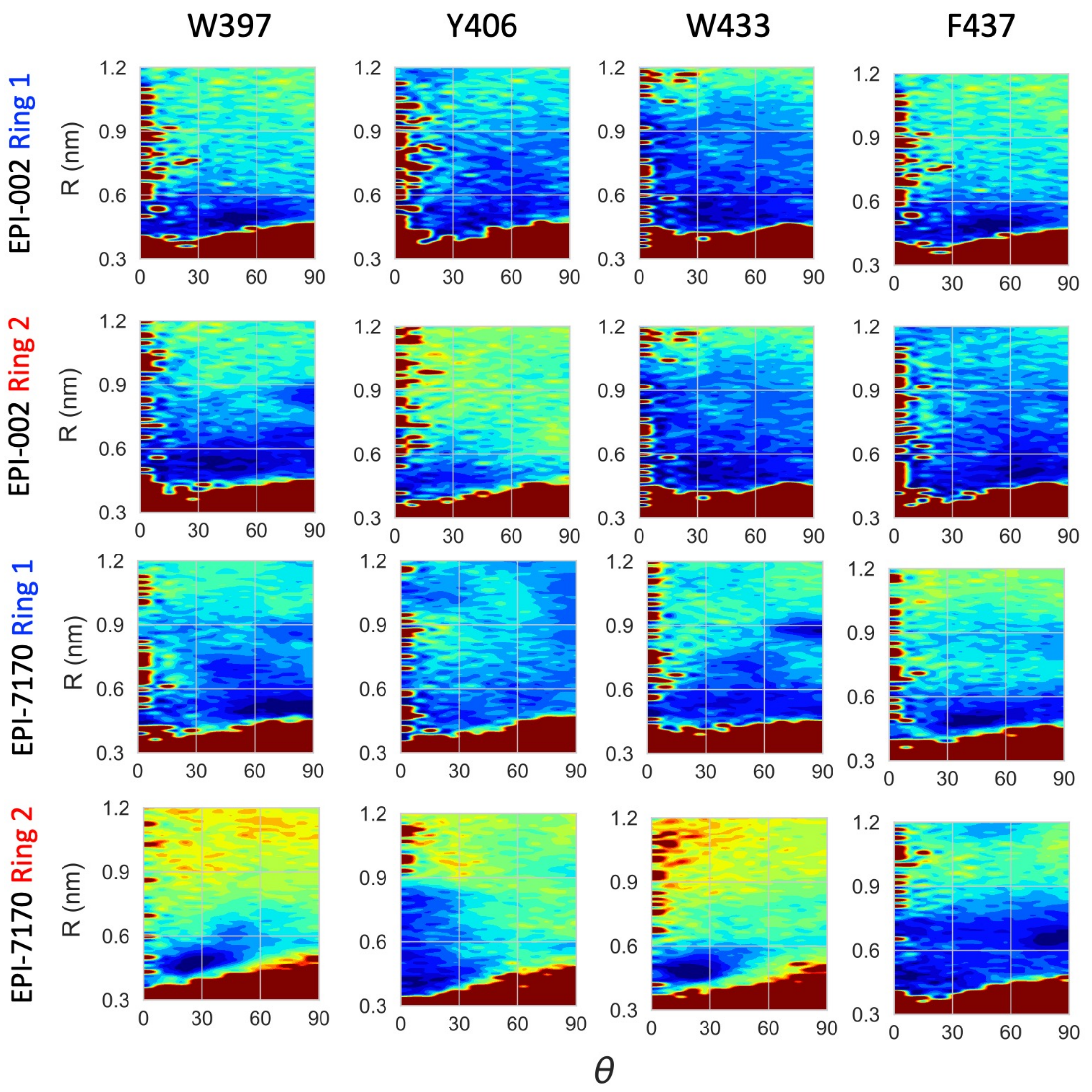

Supplementary Figure 29. Protein-ligand aromatic stacking geometries observed in the Tau-5$_{R2_R3}$:EPI-002 and Tau-5$_{R2_R3}$:EPI-7170 bound ensembles. Comparison of orientations of aromatic interactions in the EPI-002:Tau-5$_{R2_R3}$ and EPI-7170:Tau-5$_{R2_R3}$ bound ensembles. The stacking coordinate system is described in the methods section and Fig. 5 of the main text. Ring definitions are reported in Supplementary Figure 25. Free energy surfaces are shown as a function of the distance R between ring centers and the angle θ formed between the ring normal vectors

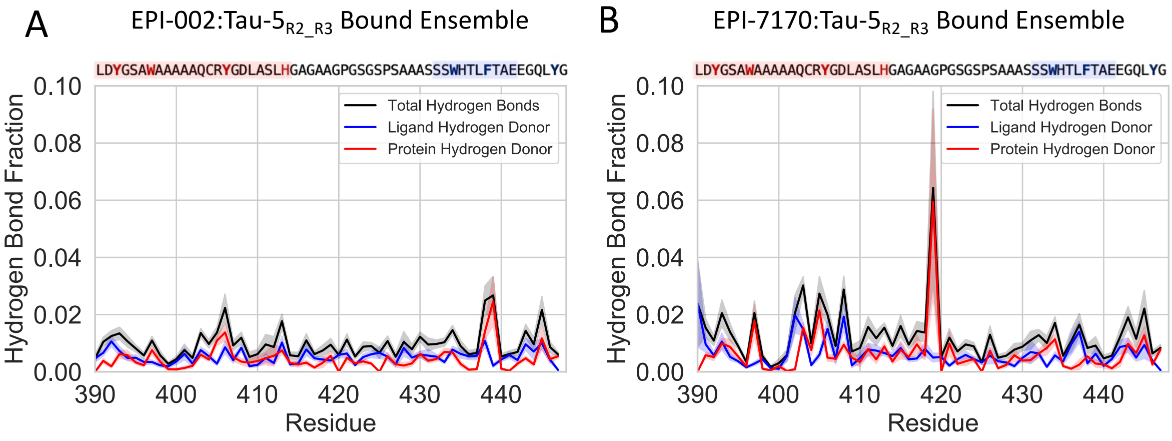

Supplementary Figure 30. Protein-ligand hydrogen bonding interactions observed in the Tau-5$_{R2_R3}$:EPI-002 and Tau-5$_{R2_R3}$:EPI-7170 bound ensembles. Comparison of intermolecular hydrogen bond populations observed in the EPI-002:Tau-5$_{R2_R3}$ (A) and EPI-7170:Tau-5$_{R2_R3}$ (B) bound ensembles. Populations are presented as mean values +/- statistical error estimates from blocking. The total fraction of hydrogen bonds for each residue are shown in black, hydrogen bonds with ligand atoms serving as hydrogen donors are shown in blue and hydrogen bonds with protein atoms serving as hydrogen donors are shown in red. We note the most populated hydrogen bond in the EPI-7170:Tau-5$_{R2_R3}$ bound ensemble (the hydrogen bond between the backbone amide of G419 and the oxygen atom in the chlorohydrin group of EPI-7170) has a large statistical error estimate (6.4$\pm$3.4%) as it is predominantly populated in a contiguous 900ns portion of the REST2 MD trajectory in a relatively narrow subset of bound conformations.

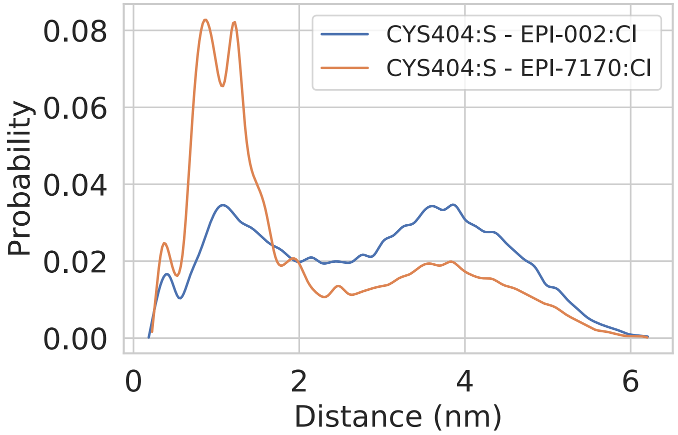

Supplementary Figure 31. Interatomic distances between the chlorohydrin groups of EPI-002 and EPI-7170 and the Tau-5$_{R2_R3}$ CYS404 thiol group. Comparison of the distribution of interatomic distances between the chlorine atom of the chlorohydrin group of EPI-002 and the sulfur atom of the the thiol group of Tau-5$_{R2_R3}$ CYS404 (blue) and the chlorine atom of the chlorohydrin group of EPI-7170 and the sulfur atom of the thiol group of Tau-5$_{R2_R3}$ CYS404 (orange) observed in the 300K solute temperature replicas of REST2 MD simulations of Tau-5$_{R2_R3}$ in the presence of EPI-002 and EPI-7170.

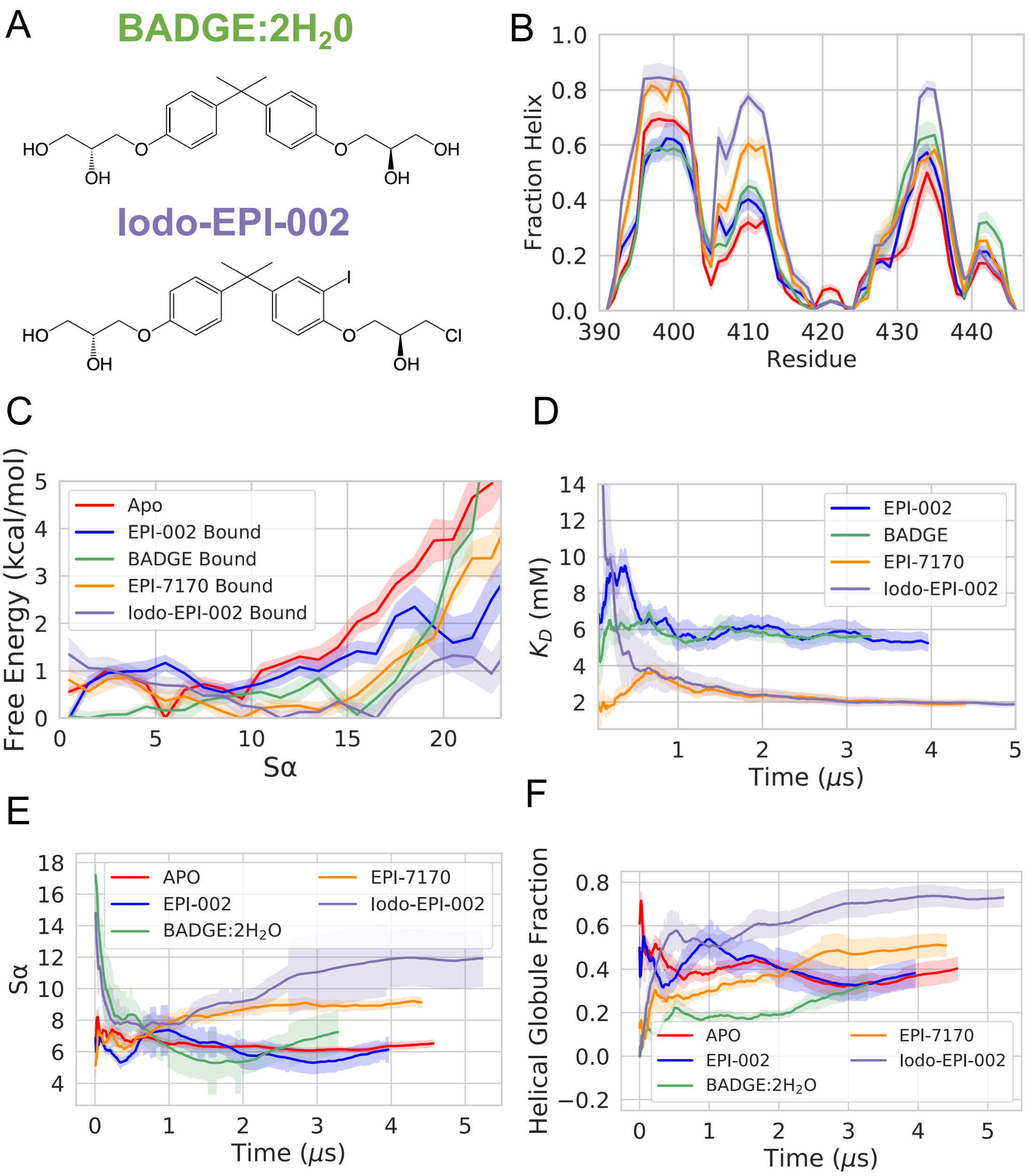

Supplementary Figure 32. Tau-5$_{R2_R3}$ REST2 MD simulations in the presence of Iodo-EPI-002 and BADGE:2H$_2$O. A) Chemical Structures of BADGE:2H$_2$O and Iodo-EPI-002. B) Helical Propensities observed in the 300K replica of explicit solvent REST2 MD simulations of Tau-5$_{R2_R3}$ in its apo form (red) and in bound conformations obtained from simulations run in presence of EPI-002 (blue), EPI-7170 (orange), BADGE:2H$_2$O (green), and Iodo-EPI-002 (purple). Simulated helical propensities are presented as mean values +/- statistical error estimates from blocking. C). Free energy surface of Tau-5$_{R2_R3}$ conformations at 300K as a function of the helical collective variable Sα for each ensemble. Free energies were calculated using the Sα values of all frames (apo) or all bound frames (ligands) from each trajectory. Shaded regions indicate the standard error of the calculated free energies when the trajectory is split into 5 equally sized blocks. D) Convergence of calculated K_D values for simulations of Tau-5$_{R2_R3}$ in the presence of ligands. E) Convergence of calculated

Sα values for simulations of Tau-5$_{R2_R3}$ in its apo form and in the presence of ligands. F) Convergence of calculated helical globule populations of Tau-5$_{R2_R3}$ in its apo form and in the presence of ligands. In panels D-F values for each time point are reported as the mean value observed in all frames of the trajectory prior to that time point +/- statistical error estimates (shaded regions). Statistical error estimates were calculated from a blocking analysis using all frames in the trajectory prior to that time point.

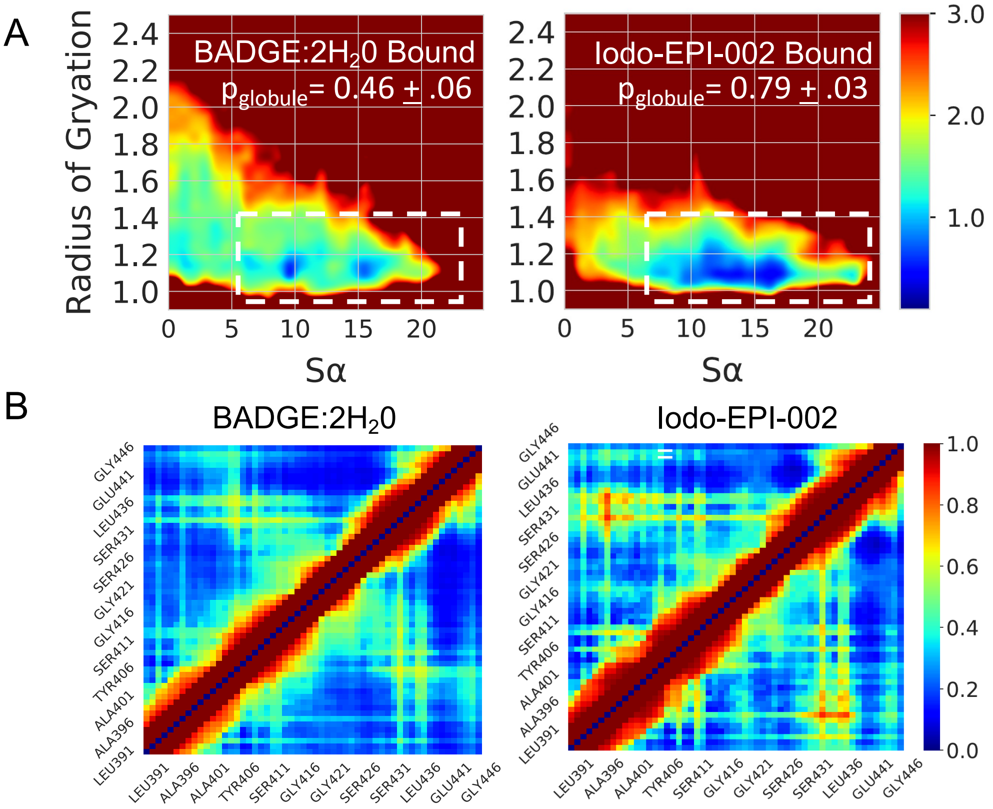

Supplementary Figure 33. Free energy surfaces and intramolecular contact maps of the Tau-5$_{R2_R3}$:BADGE:2H$_2$0 and Tau-5$_{R2_R3}$:Iodo-EPI-002 bound ensembles. A) Free energy surfaces of the BADGE:2H$_2$O:Tau-5$_{R2_R3}$ and Iodo-EPI-7170:Tau-5$_{R2_R3}$ bound ensembles as a function of radius of gyration (R_g; reported in nm) and Sα observed in the 300K solute temperature replicas of REST2 MD simulations. The dotted white lines indicate the defined boundary of "helical globule" state (Sα >6.0, R_g<1.3nm). The population of the helical globule state is reported as $p_{globule}$. B) Comparison of intramolecular contact probabilities of the 300K solute temperature replicas of REST2 MD simulations of the BADGE:2H$_2$O:Tau-5$_{R2_R3}$ and Iodo-EPI-7170:Tau-5$_{R2_R3}$ bound ensembles. Contacts between residues are defined using a cutoff distance of 12Å between Cα atoms

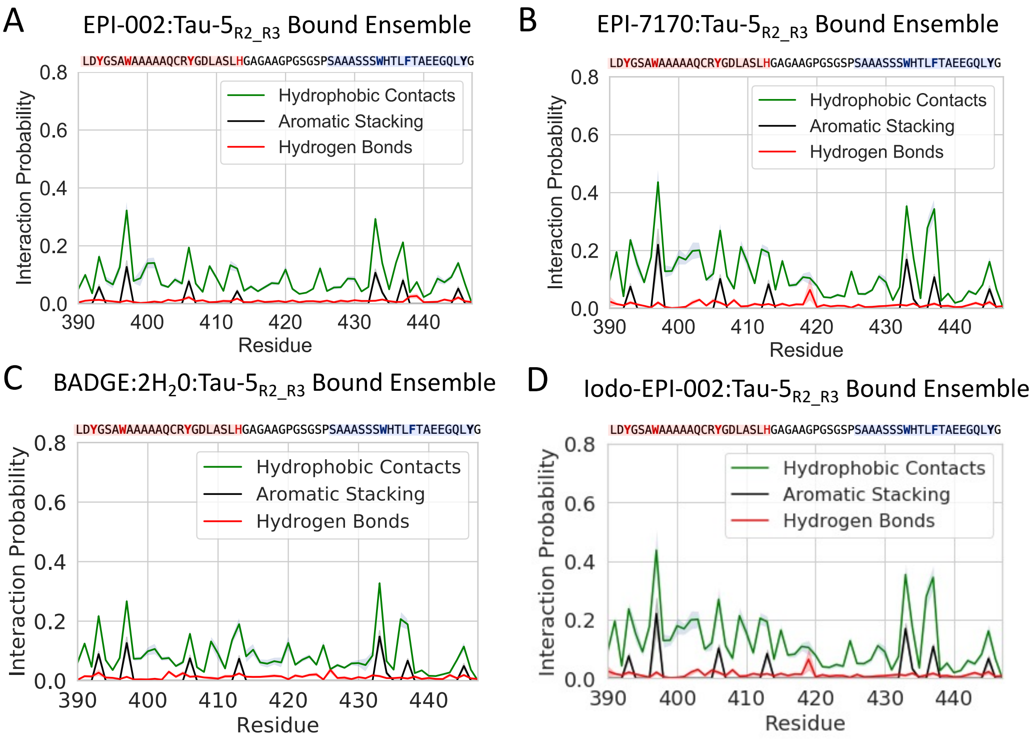

Supplementary Figure 34. Protein-ligand intermolecular interactions in the Tau-5$_{R2_R3}$ ligand bound ensembles. Populations of intermolecular interactions observed in the EPI-002:Tau-5$_{R2_R3}$ (A), EPI-7170:Tau-5$_{R2_R3}$ (B), BADGE:2H_2O:Tau-5$_{R2_R3}$ (C) and Iodo-EPI-7170:Tau-5$_{R2_R3}$ (D) bound ensembles bound ensembles. Populations are calculated considering only the bound frames of the 300K solute temperature replica of REST2 MD simulations in the presence of ligands. Populations are calculated considering only the bound frames of MD simulations in the presence of ligands. Populations of intermolecular interactions are presented as mean values +/- statistical error estimates from blocking.

	R_g (nm)	Fraction Helix	Sα	Bound Fraction	Ligand K_D (mM)	Helical Globule Population	$\Delta G_{globule}$ (kcal/mol)
ApoTau-5_{R2_R3}	1.25 ± .01	23.3 ± 0.6%	6.5 ± 0.1	-	-	40.4 ± 5.2%	+0.23 ± 0.13
EPI-002:Tau-5_{R2_R3} Bound Ensemble	1.26 ± 0.3	25.4 ± 1.3%	7.3 ± 0.4	43 ± 2%	5.24 ± 0.43	48.5 ± 6.5%	+0.04 ± 0.17
EPI-7170:Tau-5_{R2_R3} Bound Ensemble	1.24 ± .02	32.8 ± 0.5%	9.6 ± 0.2	67 ± 2%	1.92 ± 0.15	61.1 ± 4.5%	-0.27 ± 0.11
BADGE:$2H_2O$:Tau-5_{R2_R3} Bound Ensemble	1.30 ± .02	29.4 ± 1.9%	8.2 ± 0.7	44 ± 2%	4.94 ± 0.32	46.4 ± 6.0%	+0.08 ± 0.16
Iodo-EPI-002:Tau-5_{R2_R3} Bound Ensemble	1.17 ± .01	38.4 ± 1.7%	12.1 ± 0.8	67 ± 2%	1.89 ± 0.13	79.2 ± 3.3%	-0.79 ± 0.13

Supplementary Table 1. Simulated values and error estimates for properties of Tau-5_{R2_R3} in its apo form and when bound to EPI-002, EPI-7170, BADGE:$2H_2O$ and Iodo-EPI-002. Sα is an α−helical order parameter that is a proxy for the number of 6-residue helical fragments present in a protein conformation. Helical hlobule states are defined as Tau-5_{R2_R3} conformations with values of $S\alpha>6.0$ and $R_g<1.3$nm. ΔG_{glob} is the free energy of formation of the helical globule state at 300K. Values for apo Tau-5_{R2_R3} were calculated from a simulation of Tau-5_{R2_R3} performed in the absence of ligands. Values for the EPI-002:Tau-5_{R2_R3}, EPI-7170:Tau-5_{R2_R3}, BADGE:H_2O:Tau-5_{R2_R3} and Iodo-EPI-7170:Tau-5_{R2_R3} bound ensembles were calculated using only bound frames in simulations in the presence of ligands. Statistical error estimates were computed using a blocking analysis.
