# Supplementary material for: Small molecules targeting the disordered transactivation domain of the androgen receptor induce the formation of collapsed helical states
Source: Nat Commun. 2022 Oct 27;13:6390. doi: 10.1038/s41467-022-34077-z (PMC9613762; doi:10.1038/s41467-022-34077-z)
Supplement: Supplementary file 3 — Description of Additional Supplementary Files [file 41467_2022_34077_MOESM3_ESM.pdf]

Supplementary Movie 1. Conformational Ensemble of apo Tau-5$_{R2_R3}$. The conformational ensemble was obtained from the 300K replica of a 4.6μs REST2 MD simulation of apo Tau-5$_{R2_R3}$ run with the a99SB-disp protein and water forcefield. The movie was generated using a stride of 400 frames, corresponding to 32ns between frames.

Supplementary Movie 2. Conformational Ensemble of EPI-002:Tau-5$_{R2_R3}$ bound states. The bound state conformational ensemble was obtained by selecting all bound frames from the 300K replica of a 4.0μs REST2 MD simulation of Tau-5$_{R2_R3}$ in the presence EPI-002 run with the a99SB-disp protein and water force fields and the GAFF1 ligand forcefield. The movie was generate using a 400-frame stride from all bound frames

Supplementary Movie 3. Conformational Ensemble of EPI-7170:Tau-5$_{R2_R3}$ bound states. The bound state conformational ensemble was obtained by selecting all bound frames from the 300K replica of a 4.6μs REST2 MD simulation of Tau-5$_{R2_R3}$ in the presence EPI-7170 run with the a99SB-disp protein and water force fields and the GAFF1 ligand forcefield. The movie was generate using a 400-frame stride from all bound frames.
