# Supplementary material for: Small molecules targeting the disordered transactivation domain of the androgen receptor induce the formation of collapsed helical states
Source: Nat Commun. 2022 Oct 27;13:6390. doi: 10.1038/s41467-022-34077-z (PMC9613762; doi:10.1038/s41467-022-34077-z)
Supplement: Supplementary file 7 — Reporting Summary [file 41467_2022_34077_MOESM7_ESM.pdf]

nature portfolio

Corresponding author(s): Paul Robustelli

Last updated by author(s): 9/29/2022

Reporting Summary

Nature Portfolio wishes to improve the reproducibility of the work that we publish. This form provides structure for consistency and transparency in reporting. For further information on Nature Portfolio policies, see our Editorial Policies and the Editorial Policy Checklist.

Statistics

For all statistical analyses, confirm that the following items are present in the figure legend, table legend, main text, or Methods section.

n/a	Confirmed	
☒	☐	The exact sample size (*n*) for each experimental group/condition, given as a discrete number and unit of measurement
☒	☐	A statement on whether measurements were taken from distinct samples or whether the same sample was measured repeatedly
☒	☐	The statistical test(s) used AND whether they are one- or two-sided<br>*Only common tests should be described solely by name; describe more complex techniques in the Methods section.*
☒	☐	A description of all covariates tested
☒	☐	A description of any assumptions or corrections, such as tests of normality and adjustment for multiple comparisons
☐	☒	A full description of the statistical parameters including central tendency (e.g. means) or other basic estimates (e.g. regression coefficient) AND variation (e.g. standard deviation) or associated estimates of uncertainty (e.g. confidence intervals)
☒	☐	For null hypothesis testing, the test statistic (e.g. *F*, *t*, *r*) with confidence intervals, effect sizes, degrees of freedom and *P* value noted<br>*Give P values as exact values whenever suitable.*
☒	☐	For Bayesian analysis, information on the choice of priors and Markov chain Monte Carlo settings
☒	☐	For hierarchical and complex designs, identification of the appropriate level for tests and full reporting of outcomes
☒	☐	Estimates of effect sizes (e.g. Cohen's *d*, Pearson's *r*), indicating how they were calculated

Our web collection on statistics for biologists contains articles on many of the points above.

Software and code

Policy information about availability of computer code

Data collection	GROMACS 2019.270-72 patched with PLUMED v2.6.0 was used to run molecular dynamics computer simulations with the publicly distributed a99SB-disp protein, water, and ion forcefields. ACPYPE was used to generate ligand force field parameters. pmx was used to generate protein conformations for simulation starting structures
Data analysis	All analyses were performed with open source and freely dsitributed python code using the python packages MDtraj, numpy, and pyblock. All analysis code and simulation trajectories are available for download: https://github.com/paulrobustelli/AR_ligand_binding.

For manuscripts utilizing custom algorithms or software that are central to the research but not yet described in published literature, software must be made available to editors and reviewers. We strongly encourage code deposition in a community repository (e.g. GitHub). See the Nature Portfolio guidelines for submitting code & software for further information.

Data

Policy information about availability of data

All manuscripts must include a data availability statement. This statement should provide the following information, where applicable:

- Accession codes, unique identifiers, or web links for publicly available datasets
- A description of any restrictions on data availability
- For clinical datasets or third party data, please ensure that the statement adheres to our policy

The molecular dynamics trajectory data generated in this study has been deposited in Zenodo (https://doi.org/10.5281/zenodo.7120845) and is available on github (https://github.com/paulrobustelli/AR_ligand_binding). The structural ensemble of apo Tau-5R2_R3 has been deposited in the Protein Ensemble Database91 under deposition number PED00206 (https://proteinensemble.org/PED00206). The androgen receptor Tau-5 NMR chemical shifts used for trajectory reweighting are deposited in Biological Magnetic Resonance Bank entry 51479. Source data are provided with this paper.

Field-specific reporting

Please select the one below that is the best fit for your research. If you are not sure, read the appropriate sections before making your selection.

- [x] Life sciences
- [ ] Behavioural & social sciences
- [ ] Ecological, evolutionary & environmental sciences

For a reference copy of the document with all sections, see nature.com/documents/nr-reporting-summary-flat.pdf

Life sciences study design

All studies must disclose on these points even when the disclosure is negative.

Sample size	Individual long replica-exchange with solvent tempering simulations were run for each system simulated in this study. Each simulation utilized 16 independent starting structures, spanning a range of helical content, as starting structures for each solute temperature. Simulation lengths were determined based on access to computational resources and satisfactory convergence tests of simulated data. Convergence tests included reblocking analyses of simulated properties, and an analyses of the distributions of conformations sampled for each independent demultiplexed replica. Statistical error estimates and convergence analyses reported in the paper indicate satisfactory convergence of the simulated properties of interest.
Data exclusions	No data were excluded from the analyses
Replication	Replica exchange simulations inherently sample independent statistics from multiple copies of the simulated system. In our simulation set-up each replica exchange simulation contains 16 independent replicas, initiated from different starting structures, which sample the 300K canonical distribution. Statistical error estimates using reblocking analyses and convergence analyses examining the distributions of conformations sampled in each independent replica provide a quantitative assessment of the conformational sampling of each independent replica, and provide accurate error estimates on average properties computed across all 16 replicas.
Randomization	Not applicable. Randomization used in clinical trials and animal studies is usually not applicable to molecular simulations. Molecular Dynamics simulations probe specific physical systems in specific physical conditions. The algorithms used to run simulations are inherently stochastic, relying on random number generators to thermalize simulated systems and draw velocities from boltzmanm distributions. Simulated properties analyzed and compared in this manuscript were interpreted relative to simulated properties of different physical systems, with statistical error estimates provided from each simulation.
Blinding	The investigators were not blinded to results during simulations Blinding used in clinical trials and animal studies is usually not necessary in molecular simulations. All simulations in this study were analyzed using identical code, and simulated results are calculated deterministically based on this code.

Reporting for specific materials, systems and methods

We require information from authors about some types of materials, experimental systems and methods used in many studies. Here, indicate whether each material, system or method listed is relevant to your study. If you are not sure if a list item applies to your research, read the appropriate section before selecting a response.

Materials & experimental systems

n/a	Involved in the study
[x]	[ ] Antibodies
[x]	[ ] Eukaryotic cell lines
[x]	[ ] Palaeontology and archaeology
[x]	[ ] Animals and other organisms
[x]	[ ] Human research participants
[x]	[ ] Clinical data
[x]	[ ] Dual use research of concern

Methods

n/a	Involved in the study
[x]	[ ] ChIP-seq
[x]	[ ] Flow cytometry
[x]	[ ] MRI-based neuroimaging
